# Age-associated microenvironmental changes highlight the role of PDGF-C in ER^+^ breast cancer metastatic relapse

**DOI:** 10.1038/s43018-023-00525-y

**Published:** 2023-03-13

**Authors:** Frances K. Turrell, Rebecca Orha, Naomi J. Guppy, Andrea Gillespie, Matthew Guelbert, Chris Starling, Syed Haider, Clare M. Isacke

**Affiliations:** 1 The Breast Cancer Now Toby Robins Research Centre, The Institute of Cancer Research, London, UK; 2 The Breast Cancer Now Toby Robins Research Centre Nina Barough Pathology Core Facility, The Institute of Cancer Research, London, UK; 3 Flow Cytometry Facility, Chester Beatty Laboratories, The Institute of Cancer Research, London, UK

**Keywords:** Breast cancer, Cancer microenvironment, Cancer, Metastasis

## Abstract

Patients with estrogen receptor (ER)-positive breast cancer are at risk of metastatic relapse for decades after primary tumor resection and treatment, a consequence of dormant disseminated tumor cells (DTCs) reawakening at secondary sites. Here we use syngeneic ER^+^ mouse models in which DTCs display a dormant phenotype in young mice but accelerated metastatic outgrowth in an aged or fibrotic microenvironment. In young mice, low-level *Pdgfc* expression by ER^+^ DTCs is required for their maintenance in secondary sites but is insufficient to support development of macrometastases. By contrast, the platelet-derived growth factor (PDGF)-C^hi^ environment of aging or fibrotic lungs promotes DTC proliferation and upregulates tumor cell *Pdgfc* expression stimulating further stromal activation, events that can be blocked by pharmacological inhibition of PDGFRα or with a PDGF-C-blocking antibody. These results highlight the role of the changing microenvironment in regulating DTC outgrowth and the opportunity to target PDGF-C signaling to limit metastatic relapse in ER^+^ breast cancer.

## Abstract (web summary)

Turrell et al. show that, in young mice, low-level PDGF-C expression maintains dormancy of disseminated tumor cells, but, in aged or fibrotic lungs, the PDGF-C-high environment promotes proliferation, which can be counteracted with inhibition of PDGFRα or PDGF-C blockade.

## Main

Metastasis remains the cause of nearly all breast cancer-related deaths. ER^−^ breast cancers have poorer prognosis, with disease recurrence typically occurring within 5 years^1^. Conversely, although they have a better prognosis initially, patients with ER^+^ disease have a substantial risk of late recurrence and may present with metastatic disease decades later^1,2^. Underlying this extended latency period is the ability of ER^+^ DTCs to survive at the secondary site in a quiescent therapy-resistant state until triggered to re-enter proliferation and form clinically detectable metastatic lesions. The secondary-site microenvironment is key to controlling both tumor cell survival and subsequent exit from dormancy^3–11^. However, mechanistic understanding of the microenvironmental cues controlling recurrence remains limited due to the absence of suitable preclinical models of ER^+^ metastatic relapse, with the majority of studies using ER^+^ human breast cancer cells in immunocompromised mice or ER^−^ mouse mammary tumor cells. Further, given the longer latency of ER^+^ late recurrence, it is crucial to investigate metastatic relapse in models of the aging microenvironment.

In this study, we have characterized and used syngeneic models of ER^+^ breast cancer to demonstrate how microenvironmental changes due to aging or fibrotic injury support proliferation and outgrowth of DTCs. From transcriptional profiling, we identity *Pdgfc* as a key factor upregulated in aged and fibrotic lungs, associated with an accumulation of activated fibroblasts and enhanced metastatic outgrowth. By contrast, in the PDGF-C^lo^ microenvironment of young mice, expression of *Pdgfc* by disseminated ER^+^ tumor cells is required for DTC survival, highlighting the opportunity of targeting PDGF-C signaling to limit ER^+^ breast cancer recurrence.

## Results

### Syngeneic models of ER^+^ breast cancer metastatic relapse

**Fig. 1:**
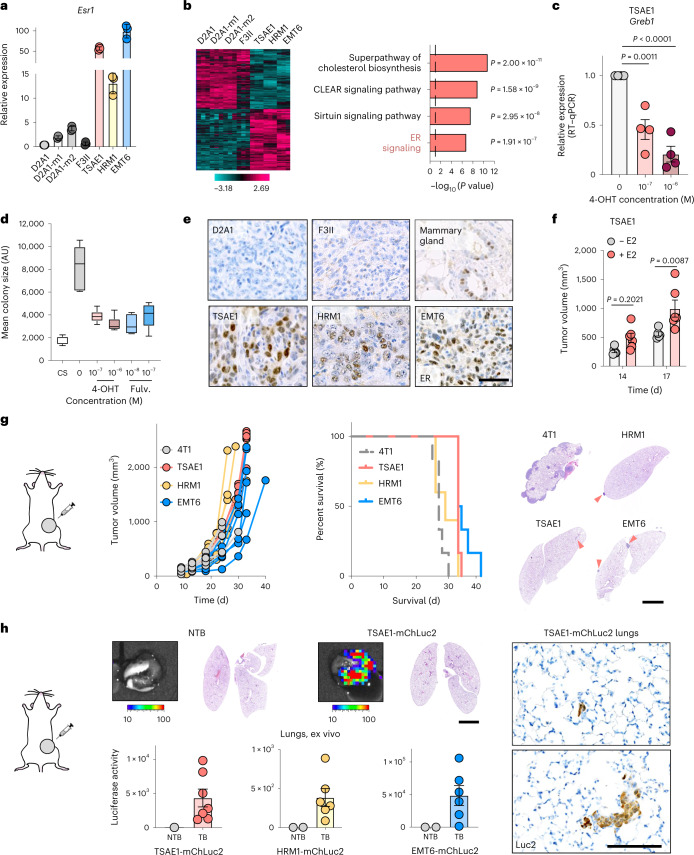
Mouse models of ER^+^ breast cancer metastatic relapse. **a**,**b**, RNA-seq analysis of mouse mammary tumor cell lines. **a**, *Esr1* expression. **b**, Left, heatmap showing significantly differentially expressed genes (|log_2_ (fold change)| > 0.585, *P* value < 0.05) between ER^−^ (F3II, D2A1, D2A1-m1, D2A1-m2) and ER^+^ (TSAE1, HRM1, EMT6) lines. Right, Ingenuity Pathway Analysis showing the top four significantly (*P* < 0.05, right-tailed Fisher’s exact test) upregulated pathways (ER^+^ versus ER^−^ lines). Dashed line, *P* value cutoff. CLEAR, coordinated lysosomal expression and regulation. **c**, *Greb1* expression in TSAE1 cells treated with vehicle or 4-OHT for 24 h (*n* = 4 biological repeats). **d**, Mean TSAE1 colony size per well (*n* = 6 wells per condition) in Matrigel following treatment with vehicle, 4-OHT or fulvestrant (fulv.) for 7 d in phenol red-free DMEM or medium with charcoal-stripped (CS) fetal bovine serum (FBS). Representative of four independent repeats. AU, arbitrary units. **e**, Primary tumors in BALB/c (TSAE1, EMT6, F3II, D2A1) or FVB (HRM1) mice stained for ER-α (scale bar, 50 µm). **f**, TSAE1 cells injected orthotopically (BALB/c) without (*n* = 5 mice) or with (*n* = 6 mice) estradiol (E2) pellets. **g**, Tumor cells injected orthotopically (TSAE1, EMT6, 4T1, *n* = 6 BALB/c mice per group; HRM1, *n* = 5 FVB mice). Left, tumor growth. Middle, survival analysis. All mice with TSAE1, HRM1 or EMT6 tumors were culled due to primary tumor size limit (solid lines); all mice with 4T1 tumors were culled due to symptoms of metastatic disease (dashed line). Right, hematoxylin and eosin (H&E)-stained lung sections (scale bar, 2.5 mm), with arrowheads indicating small metastatic deposits. **h**, mChLuc2-tagged tumor cells injected orthotopically (TSAE1, *n* = 7 and EMT6, *n* = 6 BALB/c mice; HRM1, *n* = 6 FVB mice). Mice were culled on day 33 (HRM1-mChLuc2 and EMT6-mChLuc2) or day 27 (TSAE1-mChLuc2). Top, representative lung ex vivo IVIS images (scale bar, counts) and H&E-stained lung sections (scale bar, 2.5 mm). Bottom, ex vivo IVIS quantification (total counts). Right, representative images for luciferase-stained TSAE1-mChLuc2 TB lungs (scale bar, 125 µm). **a**,**c**,**f**,**h**, Data are presented as mean values ±s.d. (**a**) or ±s.e.m. (**c**,**f**,**h**); one-way ANOVA with multiple comparisons (**c**), a box plot showing 25th and 75th percentiles, the median (line) and minimum and maximum values (whiskers) (**d**) or two-way ANOVA with multiple comparisons (**f**). Source data

**Extended Data Fig. 1:**
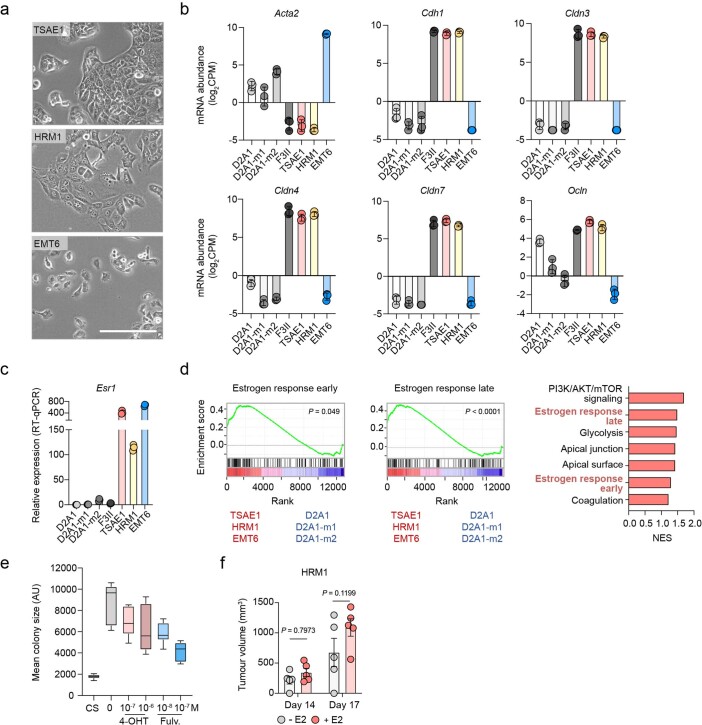
Characterisation of ER^+^ mouse mammary tumour cell lines. **a**, Phase contrast images of TSAE1, HRM1 and EMT6 cells in 2D culture (scale bar, 200 µm). **b**, Expression of *Acta2, Cdh1* and claudin genes in mouse mammary tumour lines from Fig. 1b. RNA-seq analysis (log_2_CPM). **c**, *Esr1* expression (RT-qPCR) in mouse mammary tumour cell lines with normalisation performed using average expression of four house-keeping genes (*B2m*, *Ipo8*, *18s* and *Ubc*). Representative data of four independent repeats. **d**, Associated with Fig. 1b. Left, GSEA enrichment plots for ‘Estrogen response early’ and ‘Estrogen response late’ genes comparing ER^+^ (TSAE1, HRM1, EMT6) lines (red) to ER- (D2A1, D2A1-m1, D2A1-m2) lines (blue). Right, GSEA pathways significantly (*P* < 0.05) upregulated in ER^+^ compared with ER- cell lines. NES, normalised enrichment score. **e**, Associated with Fig. 1d. Mean HRM1 colony size per well (*n* = 6 wells per condition) in Matrigel following treatment with vehicle, 4-OHT or fulvestrant (fulv.) for 7 days in phenol red-free media or in media with charcoal-stripped (CS) FBS. Representative data of two independent repeats. **f**, Associated with Fig. 1f. HRM1 cells were injected orthotopically into untreated FVB mice or mice implanted with slow release E2 pellets (*n* = 5 mice per group). **b,c,f**, Data are presented as mean values; **b**, ±SD; **f**, ±SEM, two-way ANOVA; **e**, box plot shows 25th/75th percentiles, median (line) and minimum/maximum values (whiskers). Source data

To investigate the mechanisms controlling ER^+^ breast cancer relapse, we characterized four mouse mammary tumor cell lines reported to be ER^+^ (ref. ^12^). RNA sequencing (RNA-seq) confirmed, as previously reported^12^, that EMT6 cells exhibit a claudin-low, mesenchymal phenotype, whereas claudin-expressing TSAE1, HRM1 and F3II cells display an epithelial morphology (Extended Data Fig. 1a,b). TSAE1, HRM1 and EMT6 cell lines had high *Esr1* expression, but F3II cells expressed levels comparable to those of the ER^−^ D2A1 line and metastatic derivatives D2A1-m1 and D2A1-m2 (ref. ^13^) (Fig. 1a and Extended Data Fig. 1c). ER signaling pathway genes were upregulated in TSAE1, HRM1 and EMT6 cells compared to ER^−^ cell lines, while 4-hydroxytamoxifen (4-OHT) treatment reduced expression of the ER response gene *Greb1* (Fig. 1b,c and Extended Data Fig. 1d). Similarly, the enhanced colony growth of TSAE1 and HRM1 cells in estrogen-containing medium was impaired by both 4-OHT and the selective ER degrader fulvestrant (Fig. 1d and Extended Data Fig. 1e). When grown as orthotopic tumors in syngeneic immunocompetent mice, TSAE1, HRM1 and EMT6 cells formed ER^+^ mammary tumors (Fig. 1e). Implantation of mice with estradiol pellets augmented growth of TSAE1 and, to a lesser extent, HRM1 tumors (Fig. 1f and Extended Data Fig. 1f); however, all three ER^+^ lines formed mammary tumors in mice without estrogen supplementation, hence better recapitulating the lower estradiol levels in older women.

**Extended Data Fig. 2:**
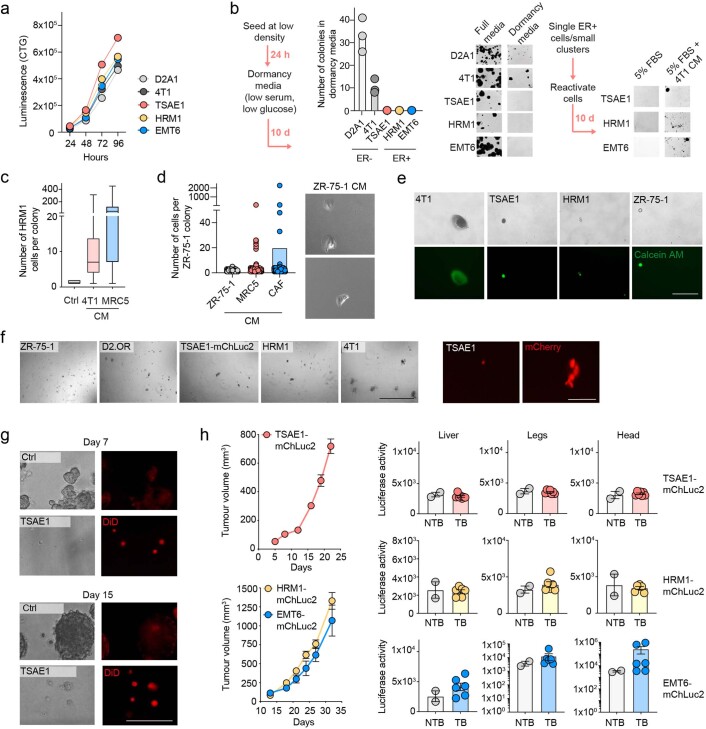
ER^+^ tumour cells exhibit dormant phenotypes. **a**, CellTiter-Glo (CTG) analysis of D2A1, 4T1, TSAE1, HRM1 and EMT6 cells. **b**, 100 tumour cells per well were seeded in 6-well plates and the following day changed into dormancy media for 10 days. Left, colonies per well (crystal violet staining), with representative images. Right, media was changed to 5% FBS or 4T1 conditioned media (CM) + 5% FBS for a further 10 days for tumour cell reactivation, representative images shown. **c**, Quantification of HRM1 tumour cells per colony following 10 days culture in Ctrl (5% FBS) or 4T1/MRC5 CM with 5% FBS (*n* = 10, 51, 35 colonies, respectively). **d**, 1,000 ZR-75-1 cells per well were seeded in 6-well plates and the following day changed into dormancy media. 7 days later CM was added as indicated for a further 10 days. Quantification of the number of ZR-75-1 cells per colony following reactivation, with representative phase contrast images of dormant ZR-75-1 cells in ZR-75-1 CM shown (scale bar, 200 µm). **e**, 5,000 cells were seeded in a soft agar-coated 6-well plates. Colonies were stained with Calcein AM 6 weeks after seeding. Representative images (scale bar, 200 µm). **f**, Representative images of tumour cells in BME dormancy assays 9 days after seeding. Scale bars; 500 µm (phase contrast), 100 µm (fluorescence). **g**, Representative images of DiD-labelled TSAE1 cells in BME dormancy assays 7 and 15 days after seeding. Ctrl, a proliferating control (higher numbers of tumour cells seeded). Scale bar, 200 µm. **h**, Additional data associated with Fig. 1h. Left, HRM1-mChLuc2, EMT6-mChLuc2 and TSAE1-mChLuc2 primary tumour growth. Right, *ex vivo* IVIS quantification of metastatic spread (total counts) to liver, hind legs and head. NTB, non-tumour bearing control mice; TB, tumour bearing. The same NTB mice were used for HRM1-mChLuc2 and EMT6-mChLuc2. Representative data of three (**a,b,f)** or two (**c,d,g)** independent repeats, with multiple cell lines. Data are presented as mean values; **h**, ±SEM; **c**, box plot shows 25th/75th percentiles, median (line) and minimum/maximum values (whiskers). Source data

In standard two-dimensional culture, the mouse ER^+^ lines grew comparably to the ER^−^ 4T1 and D2A1 lines (Extended Data Fig. 2a) but displayed a non-proliferative phenotype, remaining as single cells or small cell clusters when cultured in reduced-serum medium for 7–10 d; however, these cells could be stimulated to re-enter proliferation by subsequent culture in conditioned medium (CM) from ER^−^ 4T1 cells or normal lung fibroblasts (Extended Data Fig. 2b,c). Comparable results were observed with ZR-75-1 human ER^+^ breast cancer cells, with release from dormancy enhanced with cancer-associated fibroblast (CAF) CM compared to normal fibroblast CM (Extended Data Fig. 2d). Similarly, ER^+^ but not ER^−^ lines displayed a dormant phenotype when cultured in soft agar for 6 weeks or in growth factor-reduced basement membrane extract (BME)^4^ for 9–15 d, with cells demonstrating retention of the lipophilic dye DiD (Extended Data Fig. 2e–g). When the ER^+^ lines were injected orthotopically into syngeneic mice without supplemental estrogen, the experiment ended due to the primary tumors reaching maximum allowable size, with no evidence of ill health caused by metastatic disease. Indeed, subsequent histopathology revealed only small numbers of metastatic deposits in the lungs of some mice. By contrast, all mice bearing 4T1 tumors reached endpoint due to metastatic burden (Fig. 1g). To detect single DTCs in lungs, TSAE1, HRM1 and EMT6 cells were engineered to express mCherry and luciferase (mChLuc2). Following orthotopic implantation, organs were imaged with the In Vivo Imaging System (IVIS) ex vivo when tumors reached a size comparable to that of 4T1 tumors that cause symptomatic metastatic disease (~700–1,000 mm^3^). A positive IVIS signal was detected; however, immunostaining for luciferase revealed only single DTCs or small clusters (typically <10 cells), with no detectable macrometastatic disease in the lungs (Fig. 1h) or other organs (Extended Data Fig. 2h). Given the high ER expression and luminal classification, we focused on the TSAE1-BALB/c model, using the HRM1-FVB model for validation. No further experiments were performed with the EMT6 line due its non-epithelial, claudin-low phenotype.

### An aged microenvironment promotes outgrowth of ER^+^ DTCs

**Fig. 2:**
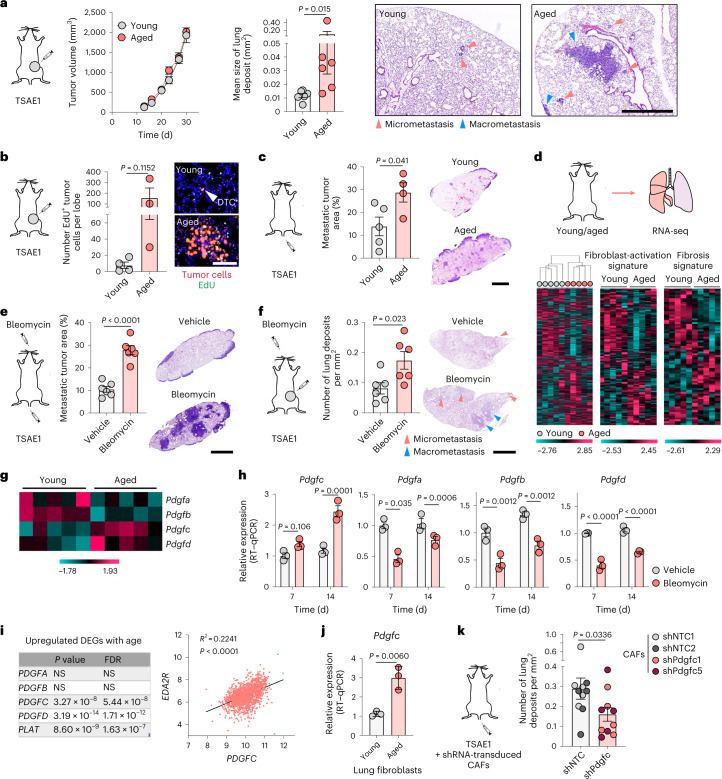
An aged microenvironment supports ER^+^ DTC metastatic outgrowth. All experiments used TSAE1 cells inoculated into BALB/c mice. **a**, Orthotopic inoculation into young or aged (15-month-old) mice (*n* = 6 mice per group). Left, tumor growth. Right, quantification of lung metastatic burden (day 30) and representative images (scale bar, 1 mm). Additional quantification is shown in Extended Data Fig. 3a. **b**, Orthotopic inoculation into *n* = 4 young or *n* = 3 aged (>12-month-old) mice. EdU was injected 24 h before culling (day 33), and EdU^+^ tumor cells in formalin-fixed paraffin-embedded (FFPE) tissue sections were quantified; representative images are shown (scale bar, 100 μm). Additional images are shown in Extended Data Fig. 3b. **c**, Intravenous inoculation into *n* = 5 young and *n* = 4 aged (9*-*month-old) mice. Metastatic lung burden (day 15), images (scale bar, 2.5 mm). **d**, RNA-seq profiling of NTB lungs from young (12-week-old) and aged (13-month-old) mice (*n* = 5 mice per group). Left, hierarchical clustering. Right, expression of ‘fibroblast-activation signature’ and ‘fibrosis signature’ genes. **e**, Intravenous inoculation into young mice 3 d after vehicle or bleomycin treatment (*n* = 6 mice per group). Metastatic lung burden (15 d after inoculation), representative images (scale, 2.5 mm). **f**, Orthotopic inoculation into young mice (day 1), treated with vehicle or bleomycin starting at day 10 (*n* = 6 mice per group). Metastatic lung burden (day 32), representative images (scale, 2.5 mm). **g**, Expression in NTB lungs of young and aged mice (RNA-seq). **h**, NTB mice treated with vehicle or bleomycin and culled 7 or 14 d later (*n* = 3 mice per group; RT–qPCR). **i**, Correlation analysis of *PDGFC* and *EDA2R* expression in human non-cancerous lung samples (*n* = 1,197 patients; GSE23546). DEG, differentially expressed gene; FDR, false discovery rate. **j**, *Pdgfc* expression (RT–qPCR) in primary fibroblasts (three independent repeats). **k**, Intravenous inoculation into young mice. On days 3 and 8, mice were injected intravenously with shNTC- or shPdgfc mouse GFP^+^ CAFs (*n* = 5 mice per group). Metastatic lung burden (day 10; shNTC and shPdgfc, *n* = 10 mice per group). **a**–**c**,**e**,**f**,**h**,**j**,**k**, Data are presented as mean values ± s.e.m.; two-tailed Mann–Whitney *U*-test (**a**,**k**), two-tailed *t*-test (**b**,**c**,**e**,**f**,**j**), two-tailed Pearson’s correlation (**i**) or two-way ANOVA with multiple comparisons (**h**). Source data

**Extended Data Fig. 3:**
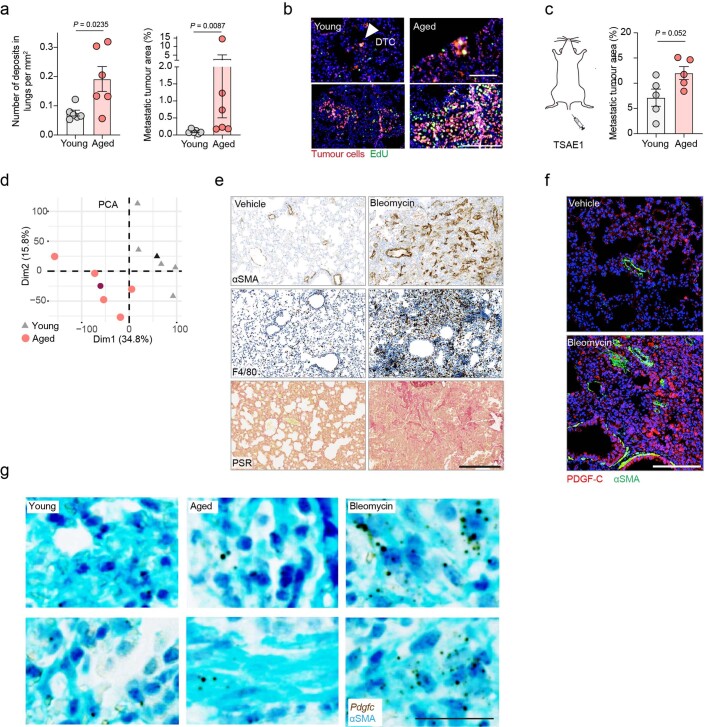
PDGF-C levels are upregulated in aged and fibrotic lungs. **a**, Additional quantification for Fig. 2a. Left, number of metastatic deposits in the lungs.^.^ Right, percentage metastatic tumour area in the lungs. **b**, Additional images of the experiment presented in Fig. 2b illustrating EdU+ tumour cells in young and aged mouse lungs. Tumour cells were identified by staining with an antibody against HMGA2. White arrowhead points to single dormant tumour cell (scale bars; 100 µm, upper images; 250 µm, lower images). **c**, Independent repeat of the experiment presented in Fig. 2c. TSAE1 tumour cells were injected intravenously into the tail vein of young (*n* = 5 mice) or aged (14-month, *n* = 5 mice) BALB/c mice. Percentage metastatic tumour burden in the lungs (day 15). **d**, Associated with Fig. 2d. Principal component analysis (PCA) of young (grey triangles) and aged (light red circles) NTB mouse lungs (RNA-seq data). Average values per group are shown with black and dark red shapes for young and aged lungs, respectively. **e,f**, BALB/c mice were treated with vehicle or bleomycin (see Methods for schedule) and culled 7 or 14 days after treatment ended (*n* = 3 mice per group). **e**, Representative images of αSMA and F4/80 (7 days post-treatment) and picrosirius red (PSR) (14 days post-treatment) staining in lungs (scale bar, 250 µm). **f**, Representative images of αSMA (green), PDGF-C (red) and DAPI (blue) staining in lungs of mice culled 14 days after treatment ended (scale bar, 150 µm). **g**, RNAscope analysis of *Pdgfc* expression in young (12-week), aged (13-month) or fibrotic (bleomycin-treated) lungs (from experiments shown in Fig. 2d,h). Lung sections were counterstained for αSMA (cyan). Scale bar, 25 µm. **a,c**, Data are presented as mean values ±SEM; **a** (left),**c**, two-tailed *t*-test; **a** (right), two-tailed Mann-Whitney *U*-test. Source data

Given the longer latency preceding ER^+^ distant recurrence, we sought to establish whether an aging microenvironment could promote outgrowth of ER^+^ DTCs. TSAE1 primary tumors grew comparably irrespective of mouse age, but only single DTCs or small tumor cell clusters were present in the lungs from young (12 weeks at endpoint) mice, whereas lungs from aged mice (16 months at endpoint) showed a significantly increased metastatic burden (Fig. 2a and Extended Data Fig. 3a). Labeling with 5-ethynyl-2′-deoxyuridine (EdU) confirmed that only a small percent of tumor cells in young lungs were proliferative, but a dramatically higher number of proliferating (EdU^+^) tumor cells were found in both small clusters and large metastatic deposits in aged lungs (Fig. 2b and Extended Data Fig. 3b). Using an experimental (intravenous tumor cell-inoculation) metastasis model, metastatic burden in all mice was greater than that observed in spontaneous models, likely due to the higher number of DTCs seeding in the lungs, albeit with metastatic lesions in young mice largely restricted to the lung edge and bronchial tree, while aged mice displayed a higher metastatic burden and a wider distribution of metastases with prominent lesions in the lung parenchyma (Fig. 2c and Extended Data Fig. 3c).

**Extended Data Fig. 4:**
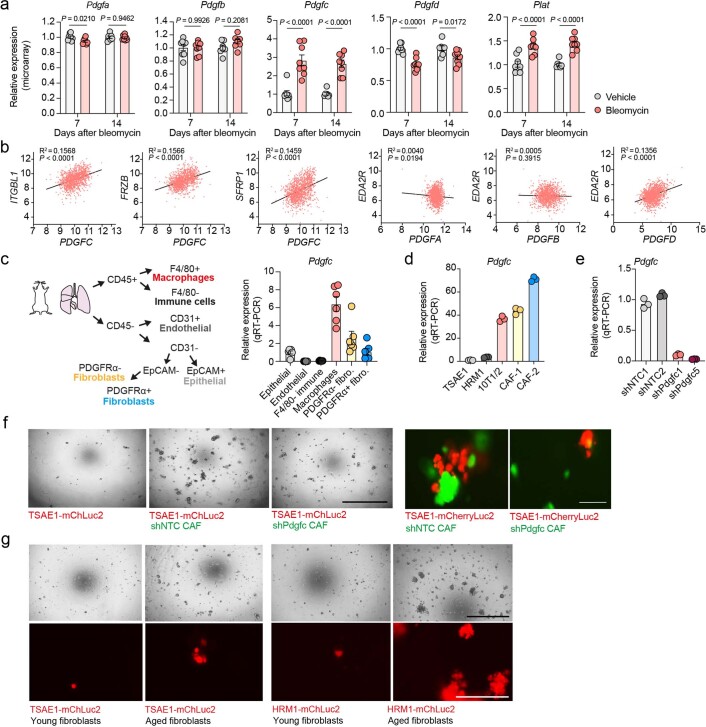
*Pdgfc*/*PDGFC* expression in the aged microenvironment and its role in release from dormancy. **a**, Transcriptional profiling of C57BL/6 mouse lungs treated with vehicle or bleomycin (from GSE40151). Analysis of *Pdgfa-d* and *Plat* expression in lungs 7 and 14 days post-treatment, *n* = 8 mice per group except *n* = 7 mice for vehicle 14 days. **b**, Associated with Fig. 2i. Transcriptional profiling of human non-cancerous lung tissues (GSE23546). Correlation of *PDGF* expression and expression of the age-associated genes *EDA2R, ITGBL1, FRZB and SFRP1*^15,17^ in lung samples from patients aged 4 − 85 years of age (*n* = 1197 patients). **c**, Expression of *Pdgfc* in the aged (18-month) C57BL/6 mouse lung (*n* = 6 mice, 4 females and 2 males). Lung tissue was dissociated and FACSorted for the following populations: macrophages (CD45^+^ F4/80^+^), other immune cells (CD45^+^ F4/80^-^), endothelial (CD45^-^ CD31^+^), epithelial (CD45^-^, CD31^-^, EpCAM^+^) and PDGFRα^-^ (cells negative for all markers) and PDGFRα^+^ fibroblasts. **d**, *Pdgfc* expression in GFP^+^ mouse CAFs compared to 10T1/2 fibroblasts and ER^+^ mouse tumour cells (RT-qPCR). **e**, GFP^+^ mouse CAFs were transduced with two non-targeting control shRNAs (shNTC1, shNTC2) or two independent shRNAs targeting *Pdgfc* (shPdgfc1, shPdgfc5). RT-qPCR to assess *Pdgfc* expression. **f**, TSAE1-mChLuc2 cells were cultured in BME assays (see Extended Data Fig. 2f) for 9 days with or without shNTC or shPdgfc CAFs and then imaged on an EVOS microscope; tumour cells (mCherry^+^) and CAFs (GFP^+^). Scale bar, 1 mm (phase-contrast images) and 100 µm (fluorescent images). **g**, TSAE1-mChLuc2 or HRM1-mChLuc2 cells were cultured in BME assays for 9 days (see Extended Data Fig. 2f) with or without immortalised young or aged mouse lung fibroblasts. Cells were imaged on an EVOS microscope: tumour cells (mCherry^+^). Scale bar, 1 mm (phase-contrast images) and 200 µm (fluorescent images). **a,c-e**, Data are presented as mean values; **a,c**, ±SEM; **a**, two-way ANOVA; **b**, two-tailed Pearson’s correlation (line shows linear regression). **f,g**, Representative of two-independent repeats. **g**, Two independent young and old fibroblast populations were used with equivalent results. Source data

RNA-seq profiles of lungs from naive young and aged BALB/c mice clustered based on age, with fibroblast activation and fibrosis genes differentially expressed (Fig. 2d and Extended Data Fig. 3d). To model lung fibrosis, we used intranasal bleomycin treatment, which promotes fibroblast activation, macrophage infiltration and enhanced collagen deposition (Extended Data Fig. 3e). Following either intravenous or orthotopic inoculation, ER^+^ TSAE1 cells showed significantly enhanced metastatic outgrowth in bleomycin-treated lungs (Fig. 2e,f), again with a notable increase in macrometastases within the lung parenchyma. Given these findings and the striking reactivation of tumor cell proliferation by fibroblast CM in our in vitro dormancy models (Extended Data Fig. 2b–d), we focused on pro-fibrotic factors that may support metastatic outgrowth in aged mice. It was intriguing to observe divergent patterns of expression of PDGF family members in the RNA-seq data, with lower expression of classical PDGFs, *Pdgfa* and *Pdgfb*, in aged mouse lungs compared to elevated expression of *Pdgfc* and *Pdgfd* (Fig. 2g). Additionally, *Pdgfc* expression and levels of PDGF-C protein were significantly upregulated in fibrotic lungs following bleomycin treatment, whereas expression of other PDGF family members was significantly downregulated (Fig. 2h and Extended Data Fig. 3f,g). These findings were validated by analysis of an independent RNA-profiling dataset of lungs from bleomycin-treated C57BL/6 mice^14^ (Extended Data Fig. 4a). In addition, in human lung tissue collected from 1,197 patients between 4 and 85 years of age^15^ expression of *PDGFC*, *PDGFD* and *PLAT* (encoding tissue plasminogen activator (tPA)), responsible for extracellular cleavage of the PDGF-C precursor^16^, was significantly correlated with age and with expression of *EDA2R* and other age-associated genes^15,17^, but there was no age-related correlation for *PDGFA* or *PDGFB* (Fig. 2i and Extended Data Fig. 4b).

In the human lung, *PDGFC* is predominantly expressed by fibroblasts and myeloid cells and, to a lesser extent, by epithelial cells^18^, findings recapitulated in flow cytometry analysis of the aged mouse lung (Extended Data Fig. 4c). Moreover, expression of *Pdgfc* is elevated in aged primary mouse lung fibroblasts compared to young fibroblasts (Fig. 2j). To determine whether the increased level of PDGF-C in fibroblasts from the aged lung promotes metastatic outgrowth of ER^+^ DTCs, PDGF-C^hi^ CAFs were transduced with non-targeting short hairpin RNA (shRNA) controls (shNTC1 and shNTC2) or shRNA species targeting *Pdgfc* (Extended Data Fig. 4d,e) and inoculated into mice 3 and 8 d after seeding of TSAE1 cells. Mice receiving *Pdgfc*-depleted CAFs displayed reduced metastatic outgrowth (Fig. 2k) and, similarly, control CAFs or fibroblasts from the aged lung but not *Pdgfc*-depleted CAFs or young fibroblasts, supported TSAE1 and HRM1 outgrowth in BME dormancy assays (Extended Data Fig. 4f,g).

### *Pdgfc* expression in the metastatic microenvironment

**Fig. 3:**
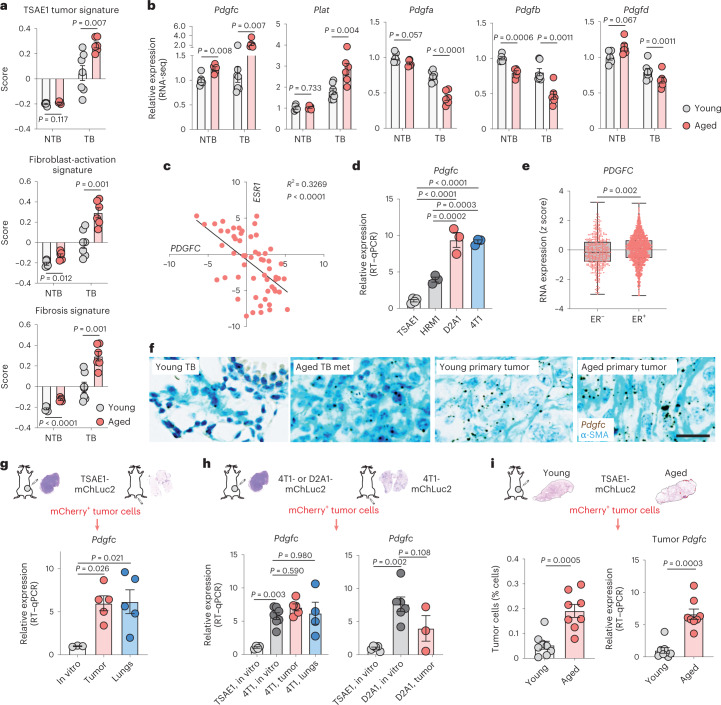
ER^+^ tumor cells contribute to elevated PDGF-C in the metastatic microenvironment. **a**,**b**, RNA-seq profiling of lungs from young or aged (13-month-old) BALB/c mice (*n* = 7 or *n* = 6 mice, respectively) inoculated orthotopically with TSAE1 cells (TB; day 29). NTB mice are from Fig. 2d. See Extended Data Fig. 5a for histology. **a**, Scores for TSAE1 tumor signature, fibroblast-activation signature and fibrosis signature (Methods). **b**, *Pdgfa*–*Pdgfd* and *Plat* expression. **c**, Correlation analysis of *ESR1* and *PDGFC* expression in human breast cancer cell lines (Cancer Cell Line Encyclopedia (CCLE)). **d**, *Pdgfc* expression (RT–qPCR) in mouse tumor lines in vitro (three independent repeats; TSAE1, four). **e**, *PDGFC* expression in human breast cancers (METABRIC dataset; ER^−^, *n* = 445 patients; ER^+^, *n* = 1,459 patients). **f**, *Pdgfc* RNAscope analysis of mouse tissue from **a**,**b**; counterstained for α-SMA (cyan). Representative images of TB young mouse lungs, metastatic deposits (met) in TB aged lungs and primary tumors (scale bar, 25 µm). **g**–**i**, mChLuc2-tagged tumor cells were isolated by flow cytometry from primary (orthotopic inoculation) and metastatic (intravenous inoculation) tumors in young mice. **g**, *Pdgfc* expression in TSAE1-mChLuc2 cells (day 16, orthotopic; day 15, intravenous; *n* = 5 mice per group) compared to cells in vitro (*n* = 3 biological repeats). **h**, Left, *Pdgfc* expression in 4T1-mChLuc2 cells from primary tumors (day 14, *n* = 5 mice), or lung metastases (day 10, *n* = 4 mice), compared to in vitro cultures (TSAE1, *n* = 4; 4T1, *n* = 7 biological repeats). Right, *Pdgfc* expression in D2A1-mChLuc2 cells from primary tumors (days 20–25, *n* = 3 mice) compared to in vitro cultures (TSAE1, *n* = 5; D2A1, *n* = 6 biological repeats). **i**, TSAE1-mChLuc2 cells inoculated orthotopically into young or aged (10–13-month-old) mice. mCherry^+^ tumor cells were isolated from the lungs (days 31–35; *n* = 8 mice per group). Left, tumor cell quantification. Right, *Pdgfc* expression in isolated tumor cells. **a**,**b**,**d**,**g**–**i**, Data are presented as mean values ± s.e.m.; multiple *t*-tests (**a**,**b**), Pearson’s correlation (two tailed) (**c**), one-way ANOVA with multiple comparisons (**d**,**g**,**h**), a box plot showing 25th and 75th percentiles, the median (line) and minimum and maximum values (whiskers) (**e**), two-tailed *t*-test (**e**,**i**, left) or two-tailed Mann–Whitney *U*-test (**i**, right). Source data

**Extended Data Fig. 5:**
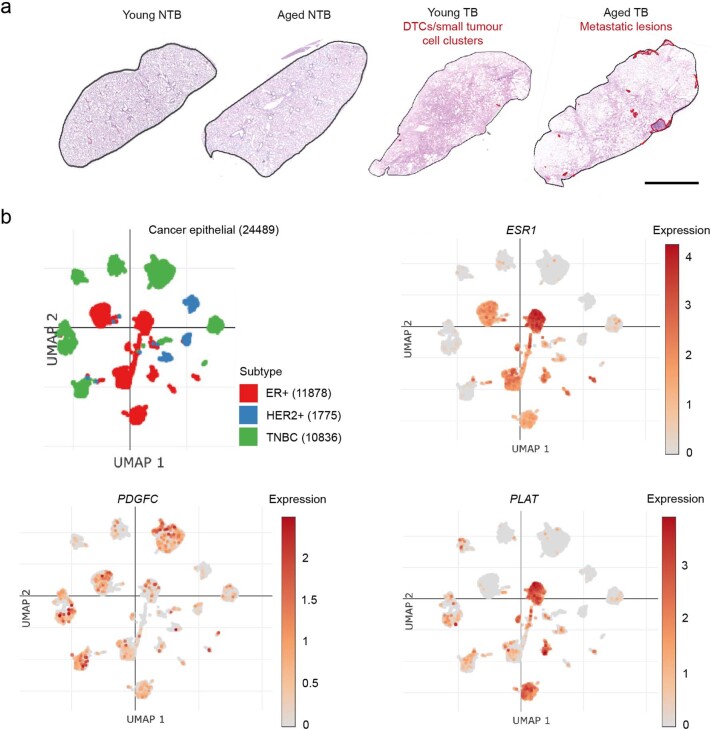
*PDGFC* expression in human breast cancer cells. **a**, Associated with Fig. 3a,b. Young (12-week) or aged (13-month) BALB/c mice were either left as non-tumour bearing (NTB, *n* = 5 mice per group) or inoculated orthotopically with TSAE1 tumour cells (TB) and culled at day 29 (*n* = 7 young mice, *n* = 6 aged mice). Lungs were divided into the left lobe for histology and the right lobes for RNA-seq analysis. Representative H&E-stained sections of left lung lobes with metastatic deposits outlined in red (scale bar, 5 mm). **b**, Single-cell RNA-seq data from 26 human breast cancers (11 ER^+^, 5 HER2^+^ and 10 TNBC, triple-negative; number of cells sequenced shown in brackets), clustering of epithelial cancer cells based on subtype^21^. Expression of *ESR1*, *PDGFC* and *PLAT* are shown. Plots were generated using the Broad Institute single-cell portal, https://singlecell.broadinstitute.org. Source data

To assess changes in the metastatic microenvironment, we compared expression of fibrosis-associated genes and PDGF family members in lungs of non-tumor-bearing (NTB) young and aged mice (from Fig. 2d) with parallel cohorts of young and aged mice inoculated orthotopically with TSAE1 tumor cells (tumor-bearing (TB) mice). Consistent with the presence of limited metastatic disease in young TB mice and macrometastatic lesions in aged TB lungs (Fig. 2a and Extended Data Fig. 5a), aged TB lungs had a higher TSAE1 tumor signature score, a pattern paralleled by increased expression of fibroblast activation and fibrosis genes, indicating further activation of the microenvironment in the presence of metastatic disease (Fig. 3a). Notably, expression of *Pdgfc* and *Plat* is further elevated in TB lungs of aged mice, whereas levels of other PDGF family members are further reduced (Fig. 3b).

In ER^−^ breast cancer, tumor cell-derived PDGF-C has been demonstrated to promote CAF recruitment^19,20^. However, the role of PDGF-C in ER^+^ breast cancer has been largely overlooked, mainly due to low expression of *PDGFC* in ER^+^ human breast cancer cell lines in vitro^20^. Indeed, expression of *PDGFC* was inversely correlated with *ESR1* expression in breast cancer cell lines (Fig. 3c), and *Pdgfc* expression was significantly lower in cultured ER^+^ TSAE1 and HRM1 tumor cells than in ER^−^ D2A1 and 4T1 cells (Fig. 3d). The situation is less clear in human tumors, with the Molecular Taxonomy of Breast Cancer International Consortium (METABRIC) dataset revealing higher *PDGFC* expression in ER^+^ breast cancers than in ER^−^ breast cancers (Fig. 3e). As METABRIC is a bulk tumor analysis, it is not possible to differentiate between tumor cell and stromal *PDGFC* expression; however, single-cell RNA-seq analysis of 26 human breast cancers^21^ demonstrated comparable *PDGFC* expression across ER^+^ and ER^−^ tumor cells, with ER^+^ cells expressing markedly higher levels of *PLAT* (Extended Data Fig. 5b). Furthermore, *Pdgfc* in situ hybridization (RNAscope) of TSAE1 TB young and aged mice revealed extensive upregulation of *Pdgfc* in primary tumors and metastatic lesions in the aged lungs (Fig. 3f), together raising the possibility that tumor cells contribute to increased *Pdgfc* expression in aged TB mouse lungs.

**Extended Data Fig. 6:**
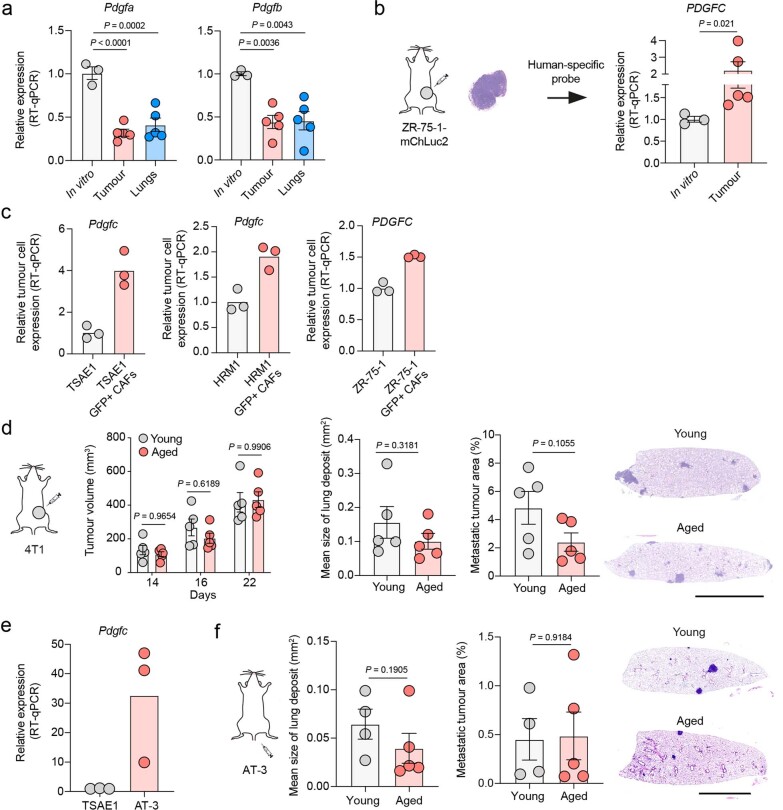
ER^-^ mouse mammary tumour cells show equivalent metastasis in young and aged mice. **a**, Additional data associated with Fig. 3g. TSAE1-mChLuc2 cells were isolated from the primary tumours or metastatic lungs, and tumour cell *Pdgfa* and *Pdgfb* expression assessed (RT-qPCR) and compared to expression in *in vitro* cultured cells (*n* = 3 biological repeats). **b**, *PDGFC* expression in ZR-75-1-mChLuc2 cells *in vitro* (*n* = 3 biological repeats) or isolated from primary tumours following orthotopic inoculation into NSG mice. RT-qPCR using a human-specific probe (*n* = 5 mice). **c**, 575,000 GFP^+^ CAFs were seeded with 200,000 TSAE1 or HRM1 tumour cells or 575,000 ZR-75-1 cells in 6-cm dishes in DMEM (10% FBS) and cultured for 48 hours. For monocultures, 400,000 TSAE1 or HRM1 cells or 750,000 ZR-75-1 cells were seeded. Single cell suspensions were stained with DAPI to exclude dead cells. 100,000-200,000 GFP-negative tumour cells were sorted on a Sony SH800 cell sorter and collected for RNA extraction. RT-qPCR analysis of *Pdgfc*/*PDGFC* expression (as detailed in Methods). Representative data of two (ZR-75-1) or three (TSAE1 and HRM1) independent repeats, with two independent CAF populations. **d**, 4T1 cells were inoculated orthotopically into young (*n* = 5 mice) or aged (>12-month, *n* = 5 mice) BALB/c mice. Left, tumour volume. Right, quantification of spontaneous metastasis to the lung, with representative images shown (scale bar, 5 mm). **e**, *Pdgfc* expression (RT-qPCR) in ER^-^ AT-3 cells culture *in vitro* compared to ER^+^ TSAE1 cells. **f**, AT-3 cells were inoculated intravenously into young (*n* = 4 mice) or aged (18-month, *n* = 5 mice) C57BL/6 mice. Quantification of lung metastasis. Data are presented as mean values; **a,b,d**,**f**, ±SEM; **a**, one-way ANOVA with multiple comparisons; **b,f** (left), two-tailed Mann-Whitney *U*-test; **d** (left), two-way ANOVA with multiple comparisons**; d**, (right),**f** (right), two-tailed *t*-test. Source data

To address this, TSAE1-mChLuc2 tumor cells were sorted from primary tumors and from lungs with metastatic lesions. Compared to cells in culture, *Pdgfc* expression was significantly elevated in ER^+^ tumor cells in vivo, whereas *Pdgfa* and *Pdgfb* were not (Fig. 3g and Extended Data Fig. 6a). Similarly, tumor cell *PDGFC*, assessed using a human-specific *PDGFC* probe, was upregulated in ER^+^ ZR-75-1 tumors compared to ZR-75-1 cells in culture (Extended Data Fig. 6b). By contrast, PDGF-C^hi^ER^−^ D2A1 and 4T1 cells did not display further upregulation of *Pdgfc* expression in vivo (Fig. 3h). Strikingly, TSAE1 cells isolated from young and aged lungs demonstrated significantly elevated tumor cell number and tumor cell *Pdgfc* expression in aged compared to young TB lungs (Fig. 3i), indicating that *Pdgfc* expression is upregulated in ER^+^ tumor cells in metastatic lesions compared to dormant DTCs. At least in part, this likely reflects the activated microenvironment associated with macrometastatic lesions as *Pdgfc* or *PDGFC* expression by ER^+^ tumor cells could be induced by co-culture with PDGF-C^hi^ CAFs (Extended Data Fig. 6c). Conversely, PDGF-C^hi^ER^−^ 4T1 and AT-3 cells did not display increased metastatic colonization in aged BALB/c and C57BL/6 mice, respectively, indicating that their high endogenous *Pdgfc* expression was sufficient to initiate metastatic colonization in the PDGF-C^lo^ microenvironment of young lungs (Extended Data Fig. 6d–f).

### Tumor cell PDGF-C supports DTC survival and outgrowth

**Fig. 4:**
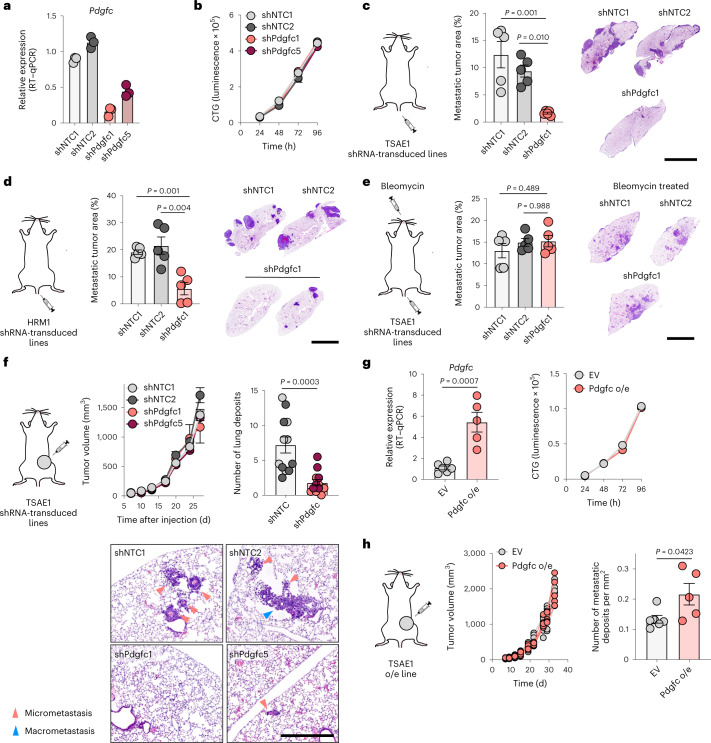
Tumor cell-derived PDGF-C in ER^+^ metastatic outgrowth. **a**, *Pdgfc* expression (RT–qPCR) in TSAE1 cells transduced with shNTC1, shNTC2, shPdgfc1 or shPdgfc5. Representative of five independent repeats. **b**, In vitro proliferation of TSAE1 cells transduced with shNTC or shPdgfc. Representative of two independent repeats. **c**, TSAE1 cells transduced with shNTC or shPdgfc were injected intravenously into young BALB/c mice (*n* = 5 mice per group). Metastatic lung burden (day 14), with representative H&E-stained sections (scale bar, 5 mm). **d**, HRM1 cells transduced with shNTC or shPdgfc (Extended Data Fig. 7a,b) were injected intravenously into young FVB mice (*n* = 5 mice per group). Metastatic lung burden (day 30). Two mice with shPdgfc tumors had no metastatic deposits. Representative H&E**-**stained sections (scale bar, 5 mm). **e**, TSAE1 cells transduced with shNTC or shPdgfc were injected intravenously into bleomycin-treated young BALB/c mice (*n* = 5 mice per group) as described in Fig. 2e. Metastatic lung burden (day 11 after TSAE1 inoculation), with representative H&E**-**stained sections (scale bar, 5 mm). **f**, TSAE1 cells transduced with shNTC or shPdgfc were injected orthotopically into young BALB/c mice (*n* = 6 mice per group). Left, tumor growth. Right, quantification of lung metastatic deposits (day 28; shNTC and shPdgfc, *n* = 12 mice per group). Bottom, representative H&E-stained sections (scale bar, 500 µm). See Extended Data Fig. 9a,b for primary tumor immunostaining. **g**, TSAE1 cells carrying control vector (EV) or expressing *Pdgfc* (Pdgfc o/e). Left, *Pdgfc* expression (RT–qPCR) in TSAE1 tumors (EV, *n* = 6 mice; *Pdgfc* overexpression, *n* = 5 mice). Right, in vitro proliferation. Representative of two independent repeats. **h**, Young BALB/c mice were injected orthotopically with TSAE1 cells carrying EV (*n* = 6 mice) or overexpressing (o/e) *Pdgfc* (*n* = 5 mice). Mice were culled on days 30–35. Left, tumor growth for individual mice. Right, quantification of lung metastatic deposits. Data are presented as mean values ± s.e.m. (**c**–**h**); one-way ANOVA with multiple comparisons (**c**–**e**) or two-tailed *t*-test (**f**–**h**). Source data

**Extended Data Fig. 7:**
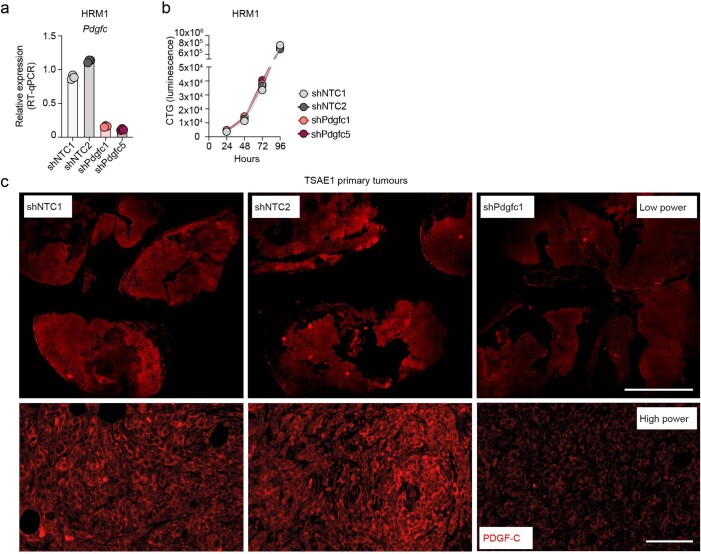
Validation of *Pdgfc* downregulated expression in HRM1 cells and TSAE1 tumours. a,b, Associated with Fig. 4d. Data are presented as mean values. a, *Pdgfc* expression (RT-qPCR) in HRM1 shNTC (1,2) and shPdgfc (1,5) cells. b, Proliferation of HRM1 shNTC (1,2) and shPdgfc (1,5) cells assessed by CellTiter-Glo (CTG). Representative data of two independent repeats. c, Immunostaining for PDGF-C in TSAE1 shNTC1, shNTC2 and shPdgfc1 primary tumours from Fig. 4f (representative images; scale bar, 5 mm and 250 µm for low- and high-power images, respectively). Source data

To test whether tumor cell-derived PDGF-C in growing metastatic lesions contributes to the development of a productive metastatic niche, TSAE1 cells were transduced with shNTC1 or shNTC2 or with two independent shRNA species targeting *Pdgfc* (shPdgfc1, shPdgfc5). All shRNA lines displayed comparable growth rate in vitro (Fig. 4a,b). As previously shown, the higher number of ER^+^ TSAE1 cells disseminating to the lung following intravenous inoculation partly overcame the deficiency of a PDGF-C^lo^ microenvironment (Fig. 2c,e), and, in these assays, *Pdgfc* knockdown significantly limited metastatic outgrowth (Fig. 4c), a finding recapitulated in the HRM1 ER^+^ model (Fig. 4d and Extended Data Fig. 7a,b). Importantly, in the bleomycin-induced lung fibrosis model, in which stromal PDGF-C levels are elevated and the lung microenvironment is extensively activated (Fig. 2h and Extended Data Fig. 3e–g), TSAE1-shPdgfc and TSAE1-shNTC cells displayed comparable metastatic outgrowth (Fig. 4e). When inoculated orthotopically, TSAE1-shNTC and TSAE1-shPdgfc primary tumors grew comparably in young mice (Fig. 4f), with immunohistochemical analysis confirming that *Pdgfc* knockdown was maintained in vivo (Extended Data Fig. 7c). As previously observed (Figs. 1g,h and 2b), young mice with TSAE1-shNTC tumors displayed evidence of dormant disseminated disease with few macrometastatic lesions. Depletion of *Pdgfc* further reduced this metastatic colonization (Fig. 4f), indicating a role for low-level tumor-derived PDGF-C in ER^+^ DTC survival. Finally, in support of the need for a PDGF-C^hi^ microenvironment for dormant DTC outgrowth, overexpression of *Pdgfc* (Fig. 4g), which had no impact on cell proliferation in vitro or primary tumor growth, significantly increased metastatic burden (Fig. 4h).

### PDGF-C-mediated fibroblast activation

**Fig. 5:**
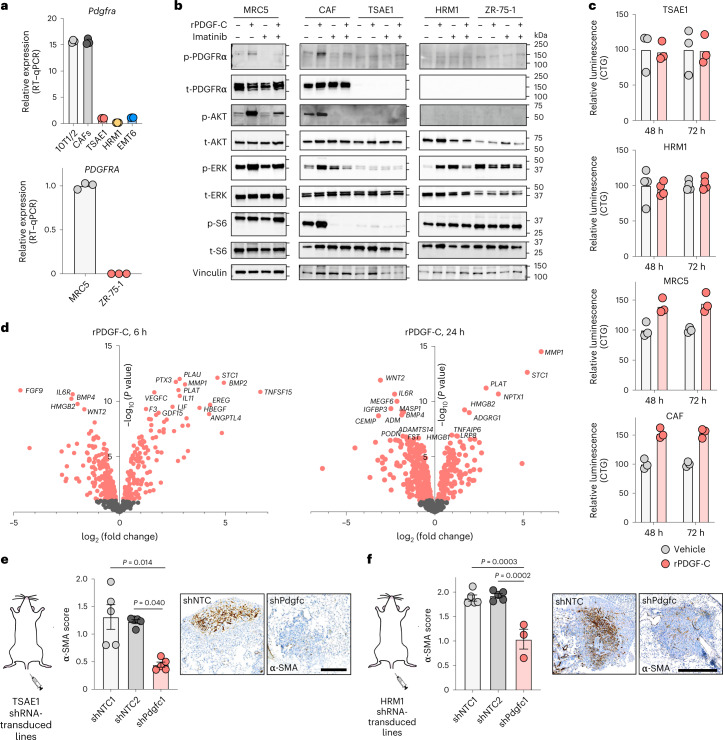
PDGF-C signals through PDGFRα to activate fibroblasts. **a**, *Pdgfra* and *PDGFRA* expression (RT–qPCR) in mouse (top) and human (bottom) fibroblasts (gray) and tumor cell lines (colored). Representative data of two independent repeats, multiple cell lines. **b**, Western blot analysis of fibroblasts and ER^+^ tumor cells following treatment with vehicle, rPDGF-C (100 ng ml^−1^) and/or imatinib (1 µM). Representative data of two independent repeats, multiple cell lines. p, phosphorylated; t, total. **c**, CellTiter-Glo (CTG) analysis of fibroblasts and ER^+^ tumor cells treated with vehicle or rPDGF-C (100 ng ml^−1^). Representative data from two (HRM1), three (TSAE1, CAF) and six (MRC5) independent repeats per cell line. **d**, RNA-seq analysis of MRC5 fibroblasts treated with vehicle or rPDGF-C for 6 h (left) or 24 h (right). Genes differentially expressed following treatment (two-tailed *t*-test, *P* < 0.01) are highlighted in red with the top 20 significantly altered genes labeled. **e**,**f**, Lungs from BALB/c mice were injected intravenously with TSAE1 tumor cells (from Fig. 4c), or FVB mice were injected intravenously with HRM1 tumor cells (from Fig. 4d), respectively, and stained for α-SMA. Metastatic deposits were scored for the number of α-SMA^+^ cells (Extended Data Fig. 9c). Representative images (scale bar, 500 µm). Note that two HRM1-shPdgfc mice had no detectable lung deposits (Fig. 4d). Data are presented as mean values; ±s.e.m., Kruskal–Wallis test with multiple comparisons (**e**,**f**). Source data

**Extended Data Fig. 8:**
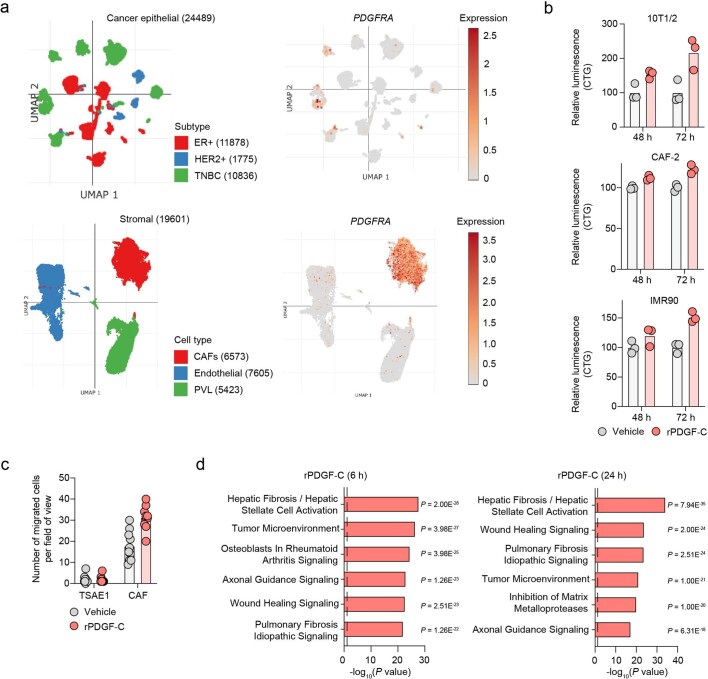
PDGF-C promotes fibroblast proliferation and migration but has no effect on tumour cells. **a**, Single-cell RNA-seq data from 26 human breast cancers (11 ER^+^, 5 HER2^+^ and 10 TNBC, triple negative)^21^. Expression of *PDGFRA* is shown in cancer and stromal cells. Number of cells sequenced is indicated in brackets. PVL, perivascular-like. Plots were generated using the Broad Institute single-cell portal, https://singlecell.broadinstitute.org. **b**, Additional data associated with Fig. 5c. CellTiter-Glo analysis following vehicle or rPDGF-C treatment (100 ng mL-^1^) for mouse (upper, middle) and human (lower) fibroblasts. Representative data of two (10T1/2 and CAF-2) or three (IMR90) independent repeats, in multiple cell lines. **c**, Transwell migration assay. Following overnight culture in SFM, 5,000 cells were seeded into Transwell inserts (8.0 µm pore size, Corning 3422) in SFM. DMEM plus 2% FBS containing vehicle or rPDGF-C (100 ng mL^-1^) was added per well. Treatments were refreshed at 24 hours. After 48 hours, inserts were washed, fixed (4% paraformaldehyde) and stained with DAPI. Non-migrated cells were removed from the top of the insert with a cotton tip. 4-5 images were taken per insert. Data show number of migrated CAF and TSAE1 cells per field of view. Representative data of three independent repeats. **d**, Ingenuity pathway analysis showing the top 6 significantly (*P* < 0.05, right-tailed Fisher’s exact test) altered pathways following treatment with rPDGF-C of MRC5 fibroblasts. Dashed line indicates *P* value cut-off. **b,c**, Data are presented as mean values. Source data

**Extended Data Fig. 9:**
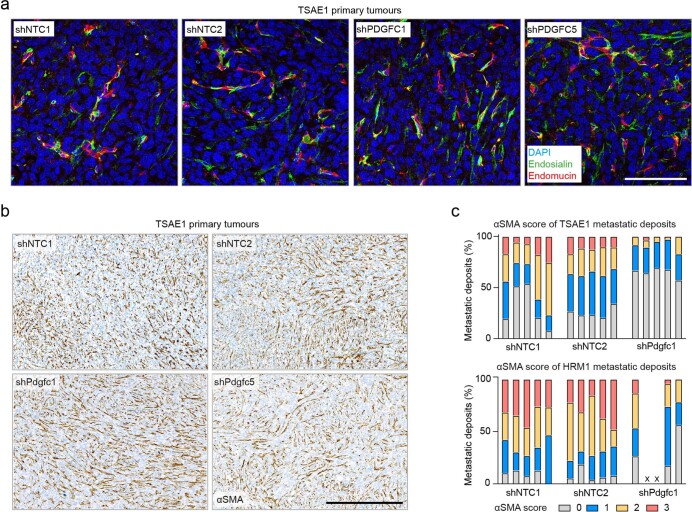
*Pdgfc* knockdown in tumour cells has limited effects on the primary tumour stroma but reduces αSMA^+^ CAF numbers in metastatic deposits. **a,b**, Representative immunostaining of shNTC (1,2) and shPdgfc (1,5) TSAE1 primary tumours from Fig. 4f. **a**, Sections stained for the endothelial marker endomucin (red), pericyte marker endosialin (green) and DAPI (blue) (scale bar, 100 µm). Brightness/contrast adjustment was performed on individual channels (ImageJ; all panels adjusted the same) before merging. **b**, αSMA immunohistochemistry (scale bar, 500 µm). **c**, Scoring of αSMA positivity of the lung metastases from Fig. 5e,f. Each bar represents an individual mouse. Scores calculated based on percentage of the metastatic deposit that was αSMA-positive: 0, 0%; 1, <20%; 2, 20-50%; 3, >50%. Note, two HRM1-shPdgfc mice had no detectable lung deposits, labelled as ‘x’. Source data

Expression of the PDGF-C receptor encoded by *PDGFRA* is restricted to fibroblasts in normal lung tissue (the Human Protein Atlas, http://www.proteinatlas.org^18^) and primary human breast cancers^21^ (Extended Data Fig. 8a). Similarly, in the ER^+^ tumor cells used here, *Pdgfra* and *PDGFRA* mRNA and PDGFRα protein levels are negligible compared to those in mouse (CAF, 10T1/2) or human (MRC5) fibroblasts (Fig. 5a,b). Indeed, recombinant PDGF-C (rPDGF-C) treatment of fibroblasts but not ER^+^ tumor cells promoted PDGFRα, AKT, ERK and S6 phosphorylation, which was suppressed by the PDGFRα inhibitor imatinib (Fig. 5b), and, consistent with previous reports^22–24^, fibroblast proliferation and migration increased following rPDGF-C treatment (Fig. 5c and Extended Data Fig. 8b,c). RNA-seq profiling of rPDGF-C-treated MRC5 fibroblasts and analysis of the secreted transcriptome identified enrichment of fibrotic and wound-healing pathways as well as increased *PLAT* expression (Fig. 5d and Extended Data Fig. 8d). PDGF-C has been reported to drive accumulation of perivascular CAFs^25^, but we observed no difference in vessel density or pericyte or perivascular CAF coverage in TSAE1 primary tumors from cells transduced with shNTC or shPdgfc (Extended Data Fig. 9a) nor the number of activated (α smooth muscle actin (α-SMA)^+^) fibroblasts (Extended Data Fig. 9b). However, there were significantly fewer α-SMA^+^ fibroblasts in shPdgfc metastatic lesions following intravenous inoculation of TSAE1 or HRM1 cells (Fig. 5e,f and Extended Data Fig. 9c), indicating that, in the young lung microenvironment, tumor-derived PDGF-C is required to promote fibroblast recruitment and activation.

### Role of PDGF-C in a human ER^+^ model of metastatic relapse

**Fig. 6:**
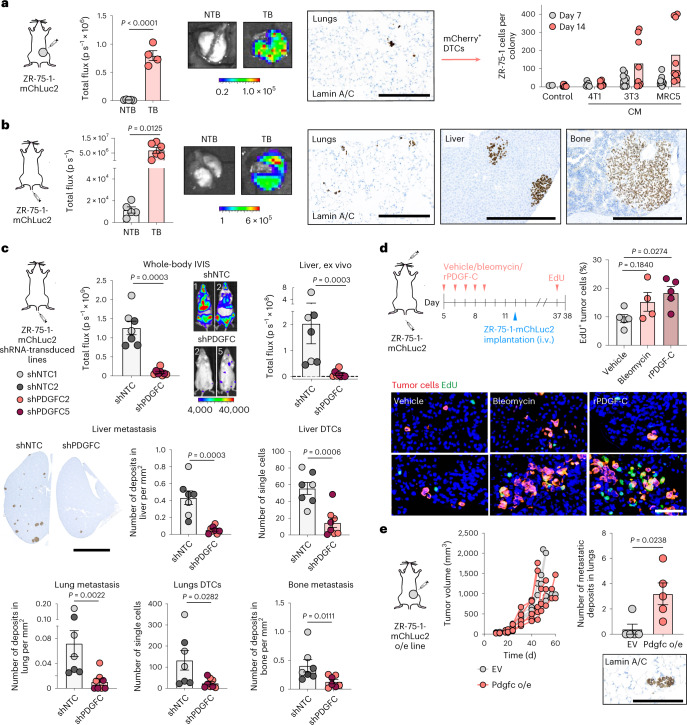
Tumor-derived PDGF-C is required for metastatic outgrowth. All experiments involved ZR-75-1-mChLuc2 cells inoculated into young NSG mice. **a**, Orthotopic inoculation (TB), with NTB controls (*n* = 5 mice per group). Left, ex vivo IVIS imaging of lungs (days 34–43; *n* = 4 TB mice as one mouse was used for tumor cell isolation; scale bar, radiance, p s^−1^ cm^−2^ sr^−1^). Right, TB lungs stained for human lamin A/C (scale bar, 250 µm). Tumor cells sorted from lungs (*n* = 1 TB mouse) were treated with control medium or 4T1 or fibroblast (MRC5 or 3T3) CM. Number of cells per colony (days 7 and 14). **b**, Intravenous inoculation (TB; *n* = 6 mice). Left*,* ex vivo IVIS imaging of lungs (day 29; scale bar, radiance, p s^−1^ cm^−2^ sr^−1^). NTB control mice were from **a**. Right, human lamin A/C staining of lungs, liver and hind leg bones (scale bars; lungs, 250 µm; liver and bones, 500 µm). **c**, Cells transduced with shNTC1, shNTC2, shPDGFC2 or shPDGFC5 were injected intravenously (shNTCs and shPDGFCs; *n* = 7 or 8 mice per group, respectively). Top left, whole-body IVIS signal (day 28; scale bar, counts). Top right, ex vivo IVIS signal in livers (day 29). Bottom left, quantification of metastatic deposits and DTCs with representative human lamin A/C-stained liver sections (scale bar, 5 mm). Only three shPDGFC5 hind leg bone samples were available. **d**, Mice were treated daily for 5 d with vehicle, bleomycin or rPDGF-C. On day 12, ZR-75-1-mChLuc2 cells were inoculated intravenously (i.v.) (*n* = 5 vehicle- or rPDGF-C-treated mice; *n* = 4 bleomycin-treated mice). Mice were injected with EdU (day 37) and culled 24 h later. Percentage of EdU^+^ human (lamin A/C^+^) tumor cells, representative images (scale bar, 50 µm). **e**, Cells carrying EV or overexpressing (o/e) *Pdgfc* cells were injected orthotopically (*n* = 5 mice per group). Mice were culled on days 45–60. Left, tumor growth for individual mice. Right, quantification of lung metastatic deposits, with representative human lamin A/C staining (scale bar, 125 µm). Data are presented as mean values; ±s.e.m. (**a**, left; **b**–**e**, right); two-tailed *t*-test (**a**–**c**, middle right), one-way ANOVA with multiple comparisons (**d**) or two-tailed Mann–Whitney *U*-test (**c**, top, middle left, bottom; **e**). Source data

**Extended Data Fig. 10:**
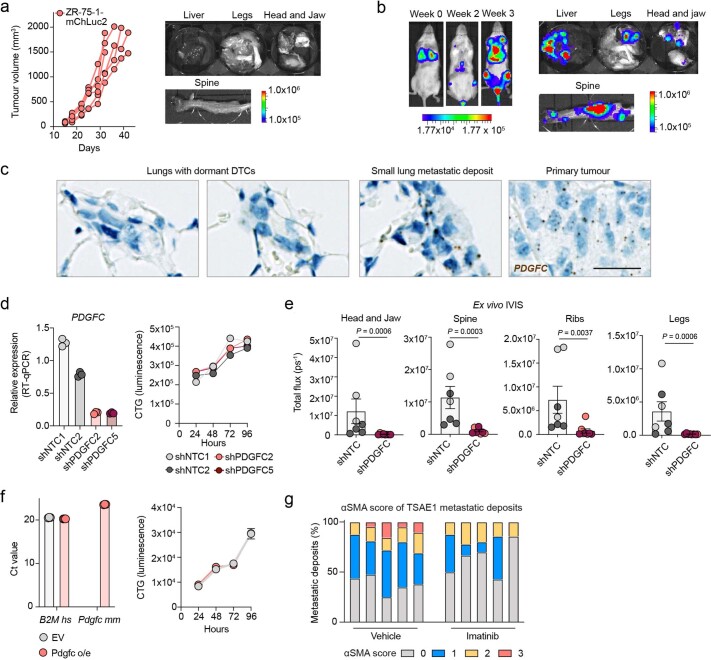
Metastasis of ZR-75-1 human ER^+^ breast cancer cells and effects of *PDGFC* knockdown or imatinib treatment. **a**, Additional data associated with Fig. 6a. ZR-75-1-mChLuc2 cells injected orthotopically into *n* = 5 NSG mice. Left, tumour growth of individual mice. Right, representative *ex vivo* IVIS images of organs at endpoint (day 34-43). **b**, Associated with Fig. 6b. ZR-75-1-mChLuc2 cells injected intravenously into *n* = 6 NSG mice. Left, representative whole-body IVIS images of one mouse at week 0 (2 hours after tumour cell inoculation), week 2 and week 3. Right, representative *ex vivo* IVIS images of organs at endpoint (day 29). **c**, *PDGFC* (human-specific) RNAscope analysis of mouse tissue with human ZR-75-1 tumour cells from Fig. 6a,b. Representative images of lungs with dormant DTCs (spontaneous, first panel; experimental metastasis, second panel), a small lung deposit (experimental metastasis) and a primary tumour. Scale bar, 25 µm. **d**, Validation of ZR-75-1 shPDGFC cell lines. Left, *PDGFC* expression (RT-qPCR) in ZR-75-1-mChLuc2 shNTC(1,2) and shPDGFC(2,5) cells cultured *in vitro*. Representative data of two independent repeats. Right, proliferation of ZR-75-1-mChLuc2 shNTC(1,2) and shPDGFC(2,5) cells assessed by CellTiter-Glo (representative data of two independent repeats). **e**, Associated with Fig. 6c. ZR-75-1-mChLuc2 shNTC(1,2) and shPDGFC(2,5) cells injected intravenously in NSG mice (shNTC(1&2) and shPDGFC5(2&5); *n* = 7 or 8 mice per group, respectively). Quantification of *ex vivo* IVIS signal in organs (day 29). Light grey, shNTC1; dark grey, shNTC2; light red, shPDGFC2; dark red, shPDGFC5. **f**, Associated with Fig. 6e. Validation of ZR-75-1 EV and Pdgfc over-expression (o/e) cell lines. Left, Ct values (RT-qPCR) for human (Hs) *B2M* and mouse (Mm) *Pdgfc* (RT-qPCR). Right, proliferation of ZR-75-1 EV and Pdgfc o/e cells assessed by CellTiter-Glo (representative data of three independent repeats). **g**, Scoring of αSMA positivity of lung metastases from Fig. 7b. Each bar represents an individual mouse. Scores calculated based on percentage of the metastatic deposit that was αSMA-positive: 0, 0%; 1, <20%; 2, 20-50%; 3, >50%. d-f, Data represent mean values; e, ±SEM, two-tailed Mann-Whitney *U*-test. Source data

To extend these studies to human breast cancer, we employed the human ER^+^ breast cancer cell line ZR-75-1. Despite being inoculated orthotopically into NOD SCID gamma (NSG) mice lacking a functional immune system, ZR-75-1 cells were found only as single DTCs or small cell clusters in the lungs with little evidence of dissemination to other organs (Fig. 6a and Extended Data Fig. 10a). Following lung dissociation, isolated ZR-75-1 cells remained non-proliferative in vitro but could be reactivated with fibroblast CM (Fig. 6a), findings consistent with in vitro dormancy assays (Extended Data Fig. 2b–d). When injected intravenously, ZR-75-1 cells disseminated to the lungs and remained largely dormant; however, tumor cells disseminating to the liver and bone, other common sites of metastasis in breast cancer, eventually formed macrometastases (Fig. 6b and Extended Data Fig. 10b). *PDGFC* RNAscope, using a human-specific probe (Extended Data Fig. 10c), confirmed quantitative PCR with reverse transcription (RT–qPCR) analysis of isolated tumor cells (Extended Data Fig. 6b), showing upregulated *PDGFC* expression in ZR-75-1 primary tumor cells but low-level *PDGFC* expression by DTCs, again supporting the contention that dormant DTCs are PDGF-C^lo^. Generation of two independent ZR-75-1-shPDGFC lines confirmed that downregulation of *PDGFC* does not impact tumor cell proliferation in vitro (Extended Data Fig. 10d) but, following intravenous inoculation, significantly reduces the number of single DTCs and larger lesions (Fig. 6c and Extended Data Fig. 10e). Conversely, pre-conditioning lungs of young mice with rPDGF-C or bleomycin promoted DTC proliferation (Fig. 6d). Together, these findings demonstrate a critical role for tumor cell-derived PDGF-C in supporting both DTC (PDGF-C^lo^) survival and metastatic (PDGF-C^hi^) outgrowth. This role of PDGF-C is further evidenced in the spontaneous metastasis setting with ZR-75-1 cells ectopically expressing *Pdgfc* disseminating to the lungs and, instead of only remaining as single dormant cells (Fig. 6a), forming metastatic deposits (Fig. 6e and Extended Data Fig. 10f).

### PDGF-C signaling blockade impairs ER^+^ metastatic outgrowth

**Fig. 7:**
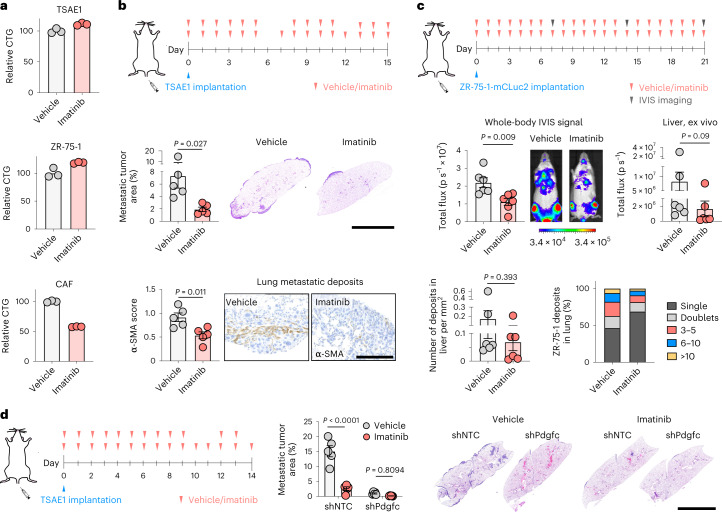
Pharmacological inhibition of PDGFRα with imatinib limits metastatic outgrowth of ER^+^ DTCs. **a**, CellTiter-Glo analysis of ER^+^ tumor cells and fibroblasts after 72 h of vehicle or imatinib (1 µM) treatment (representative data of two independent repeats, multiple cell lines). **b**, TSAE1 cells were injected intravenously into young BALB/c mice, with mice receiving vehicle or imatinib as indicated (*n* = 5 mice per group). Middle, lung metastatic burden (day 15), with representative lung sections (scale bar, 5 mm). Bottom, lungs were stained for α-SMA, and metastatic deposits were scored for the number of α-SMA-positive cells (Extended Data Fig. 10g), with representative images (scale bar, 500 µm). **c**, ZR-75-1-mChLuc2 cells were injected intravenously into young NSG mice, with mice receiving vehicle or imatinib treatment as indicated (*n* = 6 mice per group). Middle left, whole-body IVIS signal on day 14. IVIS signal quantification, with representative images (scale bar shows radiance, p s^−1^ cm^−2^ sr^−1^). Middle right, quantification of ex vivo IVIS signal in livers (day 21). Bottom left, number of liver metastatic deposits. Bottom right, percentage of tumor deposits in the lungs that are single cells, doublets, 3–5 tumor cells, 6–10 tumor cells or >10 tumor cells. **d**, TSAE1 tumor cells transduced with shNTC1 or shPdgfc1 were inoculated intravenously into young BALB/c mice, which were treated with vehicle or imatinib as indicated (*n* = 5 mice per group). Metastatic lung burden (day 14), with representative lung sections shown (scale bar, 5 mm). Data are presented as mean values; ±s.e.m. (**b**–**d**); two-tailed *t*-test (**b**,**c** (top left)), two-tailed Mann–Whitney *U*-test (**c**, top right and bottom) or two-way ANOVA with multiple comparisons (**d**). Source data

**Fig. 8:**
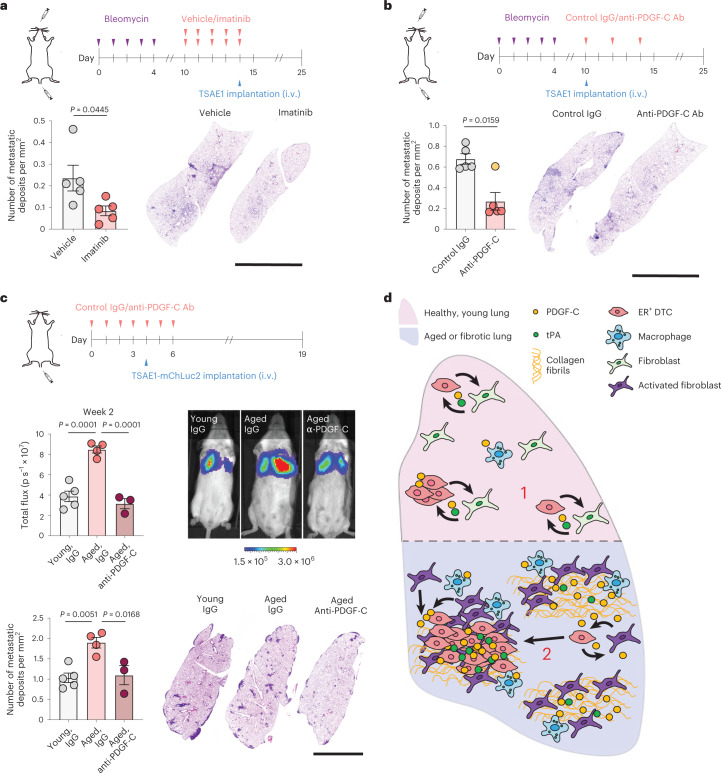
Inhibition of PDGF-C signaling in the aged or fibrotic microenvironment limits metastatic outgrowth of ER^+^ tumor cells. **a**,**b**, Young mice were pretreated with bleomycin and then received either vehicle or imatinib (**a**) or immunoglobulin (Ig)G control or anti-PDGF-C antibody (Ab) (**b**), as indicated (*n* = 5 mice per group). Mice were injected intravenously with TSAE1 cells on day 14 (**a**) or day 10 (**b**), and experiments ended on day 25. The number of lung metastatic deposits was quantified, with representative lung sections shown (scale bar, 5 mm). For **b**, an outlier was identified (yellow circle) using the outlier analysis in GraphPad Prism (ROUT method, *q* = 1%). The *P* value shown excludes the outlier (*P* = 0.0556 with the outlier included). **c**, Aged (>12-month-old) BALB/c mice were treated with control IgG (*n* = 4 mice) or PDGF-C-blocking antibody (*n* = 3 mice) as indicated. Young mice (*n* = 5 mice) were treated with control antibody. Middle, IVIS signal at week 2 after tumor cell injection, with representative whole-body IVIS images (scale bar, radiance, p s^−1^ cm^−2^ sr^−1^). Bottom, number of lung metastatic deposits, with representative lung sections (scale bar, 5 mm). **d**, Proposed model for PDGF-C in ER^+^ breast cancer metastatic relapse. (1) Single ER^+^ DTCs have low-level *PDGFC* expression, which aids DTC survival but is insufficient for the generation of a metastasis-permissive niche. (2) An activated PDGF-C^hi^ stroma in aged or fibrotic lungs supports proliferation of PDGF-C^lo^ER^+^ DTCs, with levels of PDGF-C (tumor and stroma derived) and its activator tPA elevated in the growing lesions, further activating fibroblasts and supporting the development of macrometastatic lesions. Data are presented as mean values ± s.e.m.; two-tailed *t*-test (**a**), two-tailed Mann–Whitney *U*-test (**b**) or one-way ANOVA with multiple comparisons (**c**). Source data

Given the role of tumor-derived *Pdgfc* in ER^+^ DTC survival and outgrowth, we assessed whether pharmacological inhibition of PDGFRα using imatinib limits metastatic outgrowth. As expected, given the fibroblast-restricted *Pdgfra* and *PDGFRA* expression, imatinib impacted on CAF but not tumor cell viability (Fig. 7a). Imatinib treatment of mice inoculated intravenously with TSAE1 cells suppressed metastatic outgrowth in the lungs and reduced fibroblast activation in metastatic lesions (Fig. 7b and Extended Data Fig. 10g). Similarly, imatinib treatment significantly lowered ZR-75-1 whole-body metastasis with a trend for less metastatic outgrowth in the livers and a smaller proportion of DTCs in the lungs developing into tumor cell clusters (Fig. 7c). As previously shown (Fig. 4c), *Pdgfc* depletion in TSAE1 cells severely impaired metastatic outgrowth (Fig. 7d), and, when combined with imatinib treatment, there was a small but non-significant additional reduction in lung tumor cell content, likely due, at least in part, to imatinib inhibiting PDGFRα on fibroblasts that will respond both to tumor cell- and non-tumor cell-derived PDGF-C. Finally, we sought to determine whether targeting PDGF-C signaling in PDGF-C^hi^ fibrotic and aged lungs could suppress metastatic outgrowth of ER^+^ DTCs. Treating fibrotic lungs with imatinib before TSAE1 cell inoculation or with a PDGF-C-blocking antibody, shown to block PDGF-C signaling in vivo^25,26^, from the day of TSAE1 inoculation was equally effective at reducing metastatic outgrowth (Fig. 8a,b). Similarly, the PDGF-C-blocking antibody significantly reduced the increased metastatic outgrowth observed in aged mice (Fig. 8c).

In summary (Fig. 8d), these data demonstrate that low-level *Pdgfc* expression in ER^+^ DTCs aids their survival but is insufficient for generation of a metastasis-permissive niche in healthy young lungs. Conversely, in aged or fibrotic lungs, the activated PDGF-C^hi^ stroma supports metastatic outgrowth, highlighting inhibition of PDGF-C signaling as an opportunity to limit metastatic relapse in ER^+^ breast cancers.

## Discussion

Risk of late recurrence in patients with ER^+^ breast cancer is dependent on initial clinicopathological diagnosis; however, even those with low-grade node-negative (T1N0) tumors have a 10–17% risk of distant recurrence in the following 5–20 years^2^. Despite this continuing risk, our understanding of late ER^+^ metastatic relapse remains limited by the lack of suitable preclinical models. Here, we use ER^+^ mouse mammary tumor cell lines and demonstrate that, in syngeneic young immunocompetent mice, without additional estrogen supplementation to better model the hormonal environment in older women, cells from the primary tumor disseminate to secondary sites but inefficiently develop into macrometastatic lesions. By contrast, to mimic age-associated microenvironmental changes, parallel experiments in aged mice or mice with fibrotic lungs reveal accelerated metastatic outgrowth.

Using these models, we sought to identify factors upregulated in pro-metastatic aged mouse lungs. RNA-seq analysis reveals a plethora of genes associated with fibroblast activation and development of fibrosis, previously reported to promote metastatic colonization^27^. However, of particular interest are the discordant patterns of expression of the four members of the PDGF family of growth factors. The function of PDGFs in cancers of epithelial origin has been described primarily with regard to activation of fibroblasts and/or promotion of angiogenesis^28,29^. However, few studies have examined the role of PDGFs in metastasis. Intriguingly, our studies demonstrate that, while expression of the classical PDGFs *Pdgfa* and *Pdgfb* is downregulated in aging (both NTB and TB) and fibrotic lungs, expression of *Pdgfc* and *Plat* (tPA), encoding the proteolytic enzyme required for PDGF-C activation, is strongly upregulated.

In the normal lung, liver and bone, the main sites of ER^+^ breast cancer recurrence, levels of PDGF-C are low (the Human Protein Atlas^18,30^). By comparison, in diseased tissue, increased PDGF-C levels have been reported in subsets of macrophages^25,26^, CAFs^31,32^ and tumor cells^19,33,34^. Here, we demonstrate that *PDGFC* and *Pdgfc* expression by ER^+^ cells is elevated in primary tumors and established metastatic lesions but remains low in non-proliferative DTCs at secondary sites. However, this low-level *Pdgfc* expression plays an important functional role as tumor cell *Pdgfc* depletion significantly diminishes ER^+^ DTC survival. Conversely, ectopic expression of *Pdgfc* in ER^+^ cells enhances metastatic outgrowth, indicating that the level of ER^+^ DTC-derived PDGF-C alone is insufficient for effective metastatic outgrowth. This deficiency is overcome in the PDGF-C^hi^ lung microenvironment of aged or fibrotic mice or by pre-conditioning lungs of young mice by intranasal administration of rPDGF-C. Importantly, this deficiency is not evident with PDGF-C^hi^ER^−^ 4T1 and AT-3 lines, which display equivalent metastatic outgrowth in both young and aged mice. In triple-negative breast cancers, age at diagnosis is associated with adverse prognosis with patients <40 years old having worse outcome^35^. Although other factors such as delayed detection undoubtedly contribute to poor prognosis, the prognostic effect remained significant in multivariant analysis^35^ and is further supported by mouse models, with triple-negative breast xenografts displaying earlier onset and enhanced growth in young (8–10-week-old) mice compared to their aged (>10-month-old) counterparts^36^ and the equivalent metastatic outgrowth of ER^−^ cell lines in young and aged mice as reported here.

In support of our data in the ER^+^ setting, there is a growing body of evidence describing a role for the aged stroma, including the immune cells, fibroblasts and extracellular matrix, in promoting tumorigenesis^37^. The immune compartment can regulate the balance between DTC dormancy and metastatic outgrowth^5,6,38^ and, when altered in human and mouse aging, has implications for therapy response and metastatic control^37^. In parallel, aging fibroblasts display an altered secretome, with secretion of the Wnt antagonists secreted frizzled-related protein (sFRP)1 and sFRP2 promoting metastatic outgrowth^17,39^, and, similarly, intranasal administration of transforming growth factor (TGF)-β creates a fibrotic environment, supporting outgrowth of breast tumor cells in the lungs^4^. Here, we describe upregulated expression of *Pdgfc* in aged mouse fibroblasts and a strong correlation between *PDGFC* expression and expression of age-related genes in a large dataset of human lung samples. It is important to note that the 12–16-month-old mice used here and in other studies of the aging microenvironment^17,39^ are considered equivalent to middle-aged or late middle-aged women (https://www.jax.org). As expression of age-related genes continues to increase with age in human populations^15^, it is anticipated that *Pdgfc* expression would be further elevated in 18–24-month-‘old’ mice. In addition to *Pdgfc*, we show upregulation of fibroblast activation and fibrosis genes in aged mouse lungs, and, importantly, for the majority of these, including *Pdgfc* and *Plat*, expression is further enhanced in TB lungs, supporting the positive feedback loop between tumor cells and the activated microenvironment. Although macrophages as well as fibroblasts and tumor cells contribute to this upregulation of *Pdgfc* expression in TB aged lungs, our findings are recapitulated in a dormancy model using human ER^+^ ZR-75-1 cells in immunocompromised mice, indicating that PDGF-C can elicit its pro-metastatic effects in an immune-independent manner.

Using syngeneic models of ER^+^ breast cancer in young and aged mice reveals a previously unappreciated role of PDGF-C in ER^+^ breast cancer metastasis and the utility of targeting PDGFRα with imatinib, a drug that is a well-tolerated, clinically relevant therapeutic agent with an equivalent efficacy and safety profile in old and young patients with chronic myeloid leukemia^40,41^ and can reduce bleomycin-induced fibrosis^42^. Importantly, given that imatinib additionally targets other tyrosine kinases such as KIT, CSF1R and BCR–ABL^43^, our findings are recapitulated using a PDGF-C-blocking antibody. Together, these data reinforce a growing body of evidence describing the role of the metastatic microenvironment in regulating DTC outgrowth^44–46^ and support a model in which the PDGF-C^hi^ microenvironment supports rapid metastatic outgrowth in aged mice or mice with lung fibrosis by acting to recruit and activate PDGFRα-positive fibroblasts and increase tPA levels, required for proteolytic cleavage of the PDGF-C precursor. By contrast, the low level of PDGF-C in young healthy lungs is insufficient to create a pro-metastatic microenvironment to support ER^+^ DTC outgrowth. Instead, DTC survival and outgrowth is dependent upon tumor cell-derived PDGF-C. However, it is likely that, in the clinical scenario of single ER^+^ DTCs lodged at secondary sites, as modeled here in spontaneous metastasis assays, the level of PDGF-C produced is insufficient to generate robust tumor cell–stromal cross-talk and creation of a productive metastatic niche, delaying metastatic outgrowth until these age-related changes at secondary sites trigger ER^+^ DTCs to reawaken.

## Methods

### In vivo studies

All animal work was carried out under UK Home Office Project licenses P6AB1448A and PP4856884 (establishment license X702B0E74) and was approved by the ‘Animal Welfare and Ethical Review Body’ at the Institute of Cancer Research and Imperial College London. Female BALB/c, FVB/NCrl, C57BL/6 and NSG mice were from Charles River. Young mice were 8–10 weeks old at the start of the experiment. Aged mice were aged in house or were from Charles River and were 9–18 months old at the start of the experiment (figure legends). Aged mice were closely monitored for signs of frailty. All NSG mice and, where indicated, BALB/c and FVB mice were implanted with a slow-release 17β-estradiol pellet (NE-121, 0.36 mg, 90 d; Innovative Research of America) subcutaneously 3–5 d before inoculation with tumor cells. In all cases, experiments were terminated if a mouse showed signs of ill health or lost 20% of its body weight over 72 h or if the primary tumor (unilateral) reached a mean diameter of >17 mm. The maximum tumor size permitted by the license (mean diameter, 18 mm) was not breached.

#### Orthotopic inoculation

A total of 2 × 10^5^ TSAE1, EMT6, 4T1 or D2A1 cells or 2 × 10^6^ ZR-75-1-mChLuc2 cells were injected into the fourth mammary fat pad of mice under general anesthesia. Cells were injected in sterile PBS, except EMT6 cells, which were injected in 1:1 growth factor-reduced Matrigel (Corning, 356231):PBS. Tumor growth was measured biweekly, and tumor volume was calculated using the formula volume = (length × width^2^) ÷ 2.

#### Intravenous inoculation

A total of 3–10 × 10^4^ TSAE1 or HRM1 cells, 5 × 10^5^ AT-3 cells or 3 × 10^6^ ZR-75-1-mChLuc2 cells were injected into the lateral tail vein. Mice were culled when the first mouse showed signs of metastatic disease, except for Fig. 2k, for which the experiment was ended earlier. When indicated, 5–8 × 10^5^ CAFs were injected intravenously 3 and 8 d following TSAE1 inoculation.

#### Treatments

Mice were treated with 0.1 mg per kg (NSG) or 0.25 mg per kg (BALB/c) bleomycin sulfate (Sigma, 203401) in 0.9% NaCl intranasally once daily for 5 d. BALB/c mice were dosed with 50 mg per kg imatinib mesylate (Sigma) or vehicle (water) twice daily intraperitoneally. NSG mice were dosed twice daily with 25 mg per kg imatinib mesylate or vehicle for 7 d and then 37.5 mg per kg twice daily for 2 weeks. For treatment with anti-PDGF-C-blocking antibody or normal goat IgG control, mice received 20 µg antibody in 0.9% NaCl intranasally (30 µl per nostril) every other day for three doses (Fig. 8b) or once daily for 7 d (Fig. 8c). For rPDGF-C treatment, 3 µg per 20 g mouse body weight was administered intranasally (30 µl per nostril in 0.9% NaCl) once daily for 5 d.

#### IVIS imaging

Mice were injected intraperitoneally with 150 mg per kg d-luciferin (Caliper Life Sciences) and imaged in vivo, or organs were imaged ex vivo 5 min after injection (IVIS Lumina II). Luminescence was quantified as total counts, for which imaging settings and time were kept constant, or as total flux (p s^−1^). Analysis was performed (Living Image Software, PerkinElmer, 4.7.3) maintaining the region of interest over the tissues as a constant size.

#### Quantification of metastatic burden

Two 3–4-µm lung or liver FFPE sections were cut 150–200 µm apart, H&E stained and scanned (NanoZoomer Digital Pathology (NDP), Hamamatsu). Metastatic deposits were counted (two sections per animal), with file names blinded (NDP.view2, 2.7.52). For syngeneic experiments, deposits were counted, if visible, on the H&E sections. For experiments with ZR-75-1 cells, deposits were defined when >5 human lamin A/C-positive cells were present in a cluster. For quantification of ZR-75-1 single DTCs, human lamin A/C-positive cells were counted excluding those in metastatic deposits (clusters of >5 cells). Lung or liver metastatic area was calculated manually or using Fiji (ImageJ, 2.0.0-rc-54/1.51h).

#### Immunofluorescence and immunohistochemistry

Immunohistochemical staining was performed as indicated, with detection via VECTASTAIN ABS. For immunofluorescence, 3–4-µm FFPE sections were rehydrated, antigen-retrieved (DAKO Target Retrieval Solution) and blocked in PBS (1% BSA and 2% FBS) before incubating with primary antibody overnight at 4 °C and secondary antibodies for 40 min at room temperature. Sections were scanned using NDP (low-power images). High-power images were taken with the Lecia SP8 confocal microscope. The level of gamma correction was adjusted for images on NDP and kept consistent between panels.

#### EdU-incorporation assays

EdU assays were performed as previously described^8^ using the Click-iT Plus EdU kit (Thermo Fisher Scientific, C10637). In brief, 24 h before culling, mice were injected intraperitoneally with 200 µl EdU (Thermo Fisher Scientific, E10187) solution (20 mg ml^−1^ in 0.9% NaCl). EdU^+^ tumor cells were identified in frozen sections by counterstaining with human lamin A/C (Fig. 6d) or in FFPE sections using an antibody against HMGA2 (Fig. 2b). A minimum of six images were quantified from frozen sections, or the total number EdU^+^ tumor cells was quantified in FFPE sections of one mouse lung lobe. *Hmga2* was identified as a marker to detect TSAE1 cells in the lung as it is highly expressed in TSAE1 cells (8.6 log_2_ (CPM) from RNA-seq data associated with Fig. 1a,b) but not expressed in the normal mouse lung (−2.4 log_2_ (CPM) mean expression in NTB samples from RNA-seq data from Fig. 2d), with subsequent validation of TSAE1 staining performed on tumor and lung sections.

#### RNAscope

Freshly cut 5-µm sections were used for RNAscope. IHC–ISH for α-SMA and *Pdgfc* (Mm-Pdgfc-No-XHs, ACD, 441559) was performed using the sequential co-detection workflow, or ISH was performed alone for *PDGFC* (Hs-PDGFC-No-XMm, ACD, 442919) on the Ventana DISCOVERY ULTRA (VSS version 12.5.4) with reagents from Roche and ACD (Bio-Techne) according to the manufacturer’s instructions for the RNAscope VS Universal HRP kit (ACD, 323200). A 24-min cell conditioning step and a 48-min AMP5 step were used. Control probes used were negative control (*dapB*, ACD, 312039) and positive control (human, VS probe Hs-PPIB-No-XMm, ACD, 844229; mouse, VS Probe Mm-Ppib-No-XHs, ACD, 844239). Sections were counterstained with hematoxylin and scanned on NDP.

#### Tumor cell isolation and fluorescence-activated cell sorting

Tumors and lungs were dissociated using the mouse tumor or lung dissociation kit (Miltenyi Biotec, 130-096-730 or 130-095-927), collected in Buffer S and dissociated into single-cell suspensions using the gentleMACS Octo Dissociator (Miltenyi) and the program 37C_m_TDK_2 or 37C_m_LDK_1, respectively. For isolating tumor cells, single-cell suspensions were stained with DAPI to exclude dead cells, and mCherry^+^ cells were sorted on a FACSAria III Cell Sorter directly into lysis buffer for RNA extraction using the RNeasy micro plus kit (Qiagen; Fig. 3g,h), or 1,000 ZR-75-1-mChLuc2 cells were plated per well in a 96-well plate (Fig. 6a). For isolating cell populations from aged mice (Extended Data Fig. 4c) and sorting mCherry^+^ tumor cells from young or aged TB mice (Fig. 3i), lungs were dissociated as described above into single-cell suspensions, and red blood cell lysis was performed (BD Biosciences, 555899) followed by an FC block. Cells were stained for CD45, CD31, EpCAM, PDGFRα, F4/80 and DAPI for sorting different cell populations or CD45, CD31 and DAPI to exclude immune cells, endothelial cells and dead cells, respectively. Cells were sorted on a BD Symphony S6 cell sorter directly into lysis buffer as described above, with compensation controls set up using UltraComp eBeads (eBioscience, 01-2222-42). SH800 and BD FACSDiva (8.0.1) software was used. Example gating strategies are shown in Supplementary Figs. 1 and 2.

### Reagents and cells

Antibodies and dilutions used are detailed in Supplementary Table 1. Human rPDGF-C was from Sigma (SRP3139). TSAE1 (TS/A-E1)^47^, HRM1 (ref. ^48^), EMT6 (ref. ^49^) and F3II^50^ cells were obtained from L. Wakefield^12^ (National Cancer Institute, USA) with permission of C. De Giovanni, J. Zhao, S. Rockwell and D. Alonso, respectively. D2A1 and D2.OR cells were from A. Chambers’ laboratory stocks^51^ (London Health Sciences Centre, Canada). D2A1-m1 and D2A1-m2 metastatic sublines were generated in house^13^. ZR-75-1 (CRL-1500), 10T1/2 (CCL-226), 3T3 (CRL-1658), 4T1 (CRL-2539), IMR90 (CCL-186), MRC5 (CCL-171) and HEK293T (CRL-3216) cells were from ATCC. AT-3 cells were provided by C. Paget and D. Soulard (Institute Pasteur de Lille). GFP-positive CAFs were from 4T1-TB BALB/c Ub-GFP mice^52^. Young (BALB-5013 and C57-6013) and aged (A57-6013, two independent batches from 58–78-week-old mice) mouse primary lung fibroblasts (Cell Biologics) were cultured in fibroblast medium (M2267, Cell Biologics) until immortalized (see below), after which they were cultured in DMEM (10% FBS). All other cells were maintained in DMEM (10% FBS) unless otherwise stated. All cells were routinely checked for mycoplasma contamination (MycoAlert, Lonza). The identity of ZR-75-1 cells was confirmed by short tandem repeat (GenePrint 10 System, Promega).

### In vitro studies

#### *Greb1* expression

Cells were cultured in phenol red-free DMEM (10% FBS) for >72 h before seeding. The following day, cells were treated with 4-OHT (Sigma, H7904) in phenol red-free medium (10% FBS). After 18 h, treatments were refreshed, and cells were collected for RNA extraction 6 h later.

#### CellTiter-Glo assays

Cells were seeded at 500 (mouse tumor lines) or 2,000 (fibroblasts or ZR-75-1 tumor cells) cells per well in 96-well plates. For rPDGF-C treatment, cells were serum starved the following day for 6 h before being treated with vehicle or 100 ng ml^−1^ rPDGF-C in serum-free DMEM (SFM) for 48 or 72 h. For imatinib treatment, the following day, cells were treated with vehicle or 1 µM imatinib mesylate for 72 h. Cell viability was analyzed by CellTiter-Glo (Promega) at the indicated time points.

#### Colony-formation assays

Cells were cultured in phenol red-free DMEM (10% FBS) for >72 h before seeding. A total of 10,000 cells were seeded in 100 µl 1:1 Matrigel (356237):phenol red-free DMEM (10% FBS). One milliliter of phenol red-free DMEM (10% FBS) was added with vehicle, 4-OHT or fulvestrant (Sigma, 14409) at the indicated concentrations in duplicate. For charcoal-stripped conditions, cells were seeded in and subsequently treated with phenol red-free DMEM with 10% charcoal-stripped FBS (Gibco, 12676029). Treatments were refreshed on day 4 after seeding. On day 7, a minimum of three images were taken per well (six wells per treatment group). Colony size was calculated using ImageJ.

#### Dormancy and reactivation assays

Cells were seeded at 100 (mouse lines) or 1,000 (ZR-75-1) cells per well in six-well plates in DMEM (10% FBS). The following day, the medium was changed to dormancy medium (DMEM plus 2% FBS, 4.5 g l^−1^ glucose for ZR-75-1 cells and 1 g l^−1^ glucose for mouse tumor cells). After 7–10 d, multiple images were taken per well on the EVOS microscope, and wells were stained with crystal violet. The remaining wells were refreshed with dormancy medium or treated with DMEM or CM plus 5% FBS. Seven to 10 d later, wells were stained and imaged. For generation of CM, tumor cells or fibroblasts were grown to 70–80% confluency, washed once and cultured in SFM. CM was collected 24 h later, centrifuged at 300*g* and filtered through a 0.2-µm filter. CM was supplemented with FBS after collection as indicated. For soft agar assays, 1.5 ml of 1:1 1% agar:medium (10% FBS) was plated per well of a six-well plate and allowed to set. A total of 5,000 cells were seeded in 1.5 ml of 1:1 0.6% agar:medium (10% FBS). Every 3–4 d, 200 µl medium was added. After 6 weeks, live cells were stained with 4 µM calcein AM for 1 h at room temperature and imaged on an EVOS microscope. BME assays were performed as described previously^4^, with 800 tumor cells seeded per well in a 96-well plate or 2,000 cells in a chamber slide well, or, for co-cultures, with the addition of 1,000 or 2,500 fibroblasts, respectively. Immortalized primary lung fibroblasts were used (see below). Images were taken every 3–4 d for 3 weeks. For DiD staining, 1 × 10^6^ tumor cells were incubated at 37 °C for 20 min in the dark with 5 µl Vybrant DiD Cell-Labeling Solution (Thermo Fisher Scientific, V22887) in 1 ml SFM, washed and seeded in the BME assays as described above. For proliferative controls, 8,000 (96-well) or 20,000 (chamber slide well) DiD-labeled tumor cells were seeded. For the experiment shown in Fig. 6a, ZR-75-1-mChLuc2 cells isolated from lungs were cultured in DMEM (10% FBS) for 3 d, before the medium was changed to fresh DMEM (5% FBS) or 4T1, 3T3 or MRC5 CM supplemented with 5% FBS. Images were taken after 7 and 14 d, and the number of tumor cells in each colony was counted.

#### Viral production and infection

shRNA plasmids were purified from MISSION TRC shRNA bacterial glycerol stocks (Sigma; Supplementary Table 2). The PGK-H2BmCherry-IRES-Luc2 plasmid was used for the generation of mChLuc2-tagged cell lines^52^. The EX-Mm07346-Lv185 *Pdgfc* plasmid and empty control vector (EV; EX-NEG-Lv185) were from GeneCopoeia. All plasmids were packaged using HEK293T cells and helper plasmids pRRE, pREV and pVsV-G using standard protocols. Viral medium was collected 48 h after transfection, and target cells were infected with the viral medium supplemented with 8 µg ml^−1^ polybrene. Primary lung fibroblasts were immortalized using an HPV-16 E6/E7 lentivirus (1 × 10^6^ IU ml^−1^, NBS Biologicals, G268; EF1α promoter). Virus-containing medium was added at a 1:1 dilution with medium to fibroblasts and incubated with 8 μg ml^−1^ polybrene for 48 h. All transduced cells were subsequently selected with puromycin (stable knockdown or overexpression lines) or G418 (fibroblasts) or by sorting for mCherry^+^ cells (mChLuc2 cell lines).

#### Western blotting

Cells were serum starved overnight and then pretreated with vehicle or imatinib mesylate (1 µM) for 1 h in SFM before being treated with vehicle or 100 ng ml^−1^ rPDGF-C in the presence of vehicle or 1 µM imatinib mesylate for 30 min (SFM). Cells were lysed in RIPA buffer (Sigma), and blots were blocked with 5% milk before incubation with antibodies. Blots were imaged and analyzed on a ChemiDoc System with Image Lab (Bio-Rad, 6.1).

### Quantitative PCR with reverse transcription

*Pdgfc* expression was assessed in primary lung fibroblasts before immortalization. Lungs or tumors were homogenized in lysis buffer using a Precellys tissue homogenizer. RNA was isolated using Qiagen RNeasy Plus Micro (homogenized tissues) or RNeasy Plus Mini (cells) kits. cDNA was generated using the QuantiTect reverse transcriptase kit (Qiagen) or the SuperScript IV kit (Invitrogen) with random hexamers for overexpression lines. RT–qPCR was performed with Taqman Gene Expression Assay probes (Supplementary Table 3) on a QuantStudio 6 Flex Real-Time PCR System (Applied Biosystems, 1.7.1). Each reaction was performed in triplicate, and relative expression levels were normalized to *B2m* or *B2M* and/or *Ipo8* or *IPO8*. When calculating the relative expression across independent cell lines, multiple house-keeping genes were used (*B2m*, *Ipo8*, *Ubc* and *18s*).

### RNA-seq

RNA was isolated from mouse mammary tumor cell lines or rPDGF-C- or vehicle-treated MRC5 fibroblasts or from the right lung of mice using the Qiagen RNeasy kit. Quality and quantity of the RNA was assessed using a Bioanalyzer and Qubit. Sequencing was performed using the Illumina HiSeq 4000 (in vivo RNA-seq) or the NovaSeq 6000 (RNA-seq of cell lines). RNA-seq generated 13.7–111 million reads per sample. Library quality was evaluated using FastQC (version 0.11.4), and initial bioinformatic analysis and data normalization were performed as previously described^52^.

For expression analysis of mouse mammary tumor cells in vitro (Fig. 1), genes with |log_2_ (fold change)| > 0.585 and *P* value < 0.05 between ER^+^ (TSAE1, HRM1 and EMT6) and ER^−^ (D2A1, D2A1-m1 and D2A1-m2 and F3II) cell lines were considered statistically significant. Analysis of differentially expressed genes (*P* < 0.05) in ER^+^ versus ER^−^ cell lines was performed with Ingenuity Pathway Analysis (version 01-20-04), and heatmaps were generated with MeV (4.8.1). Gene set enrichment analysis (GSEA 4.1.0) was performed on ER^−^ (D2A1, D2A1-m1 and D2A1-m2) and ER^+^ (TSAE1, HRM1 and EMT6) groups with a minimum gene set size of 15 genes and 1,000 permutations. Pathways with a normalized enrichment score (NES) > 0 and *P* value < 0.05 were considered upregulated, and those with NES < 0 and *P* value < 0.05 were considered downregulated. For RNA-seq analysis of young and aged mouse lungs (Figs. 2d,g and 3a,b), principal-component analysis was performed using R packages FactoMineR (version 2.4) and factoextra (version 1.0.7). The TSAE1 tumor signature (Supplementary Table 4) comprises a list of 33 genes that are not expressed in normal NTB mouse lungs (average log_2_ (CPM) < −2) but are expressed by TSAE1 cells in vitro (average log_2_ (CPM) > 2.9) as well as *Krt14* and *Krt16*, which exhibit the same pattern and are used to assess malignant cells in bulk tissue^53^. The fibroblast-activation signature (151 genes; Supplementary Table 5) was compiled from genes in fibroblast-activation, -proliferation and -migration signatures from the Molecular Signatures Database (GSEA; downloaded January 2021), in addition to fibroblast-activation markers *Acta2*, *S100a4* and *Fap*. The fibrosis signature (Supplementary Table 6) comprises 75 genes present in two or more of four published fibrosis datasets and the mouse Fibrosis V2 Panel (NanoString)^54–56^. Signature scores were estimated using the singular value decompositions on scaled data of constituent genes in each signature.

For PDGF-C response genes, initial analysis was performed as described above. Reads were trimmed using trim-galore version 0.6.6 and aligned to human genome assembly GRCh38 using STAR aligner version 2.7.6a. Raw counts were normalized using the R package edgeR’s (version 3.28.1) TMM (trimmed mean of *M* values) method in the R statistical programming environment (version 3.6.0). Low-abundance genes were removed using edgeR’s function ‘filterByExpr()’. Differential mRNA-abundance analysis was performed using the model: ‘~0 + group’ followed by edgeR’s quasi-likelihood *F*-test. Analysis focused on 1,903 genes predicted to have a secreted protein product (the Human Protein Atlas).

### Human dataset analysis

For analysis of the METABRIC dataset^57,58^, 1,904 human breast tumors for which ER status was known were used for the assessment of *PDGFC* expression. Plots of single-cell RNA-seq data from 26 human breast cancers^21^ were generated using the Broad Institute Single Cell Portal, https://singlecell.broadinstitute.org. For further details, see Data availability.

### Statistics and reproducibility

No statistical method was used to predetermine sample size, but sample sizes are similar to those previously reported^13,52,59^. Data collection and analysis were not performed blinded to the conditions of all experiments. For in vitro studies, no randomization was performed, and experiments were repeated at least twice and/or validated with independent cell lines. Representative experiments were repeated, as indicated, with similar results. Age-matched mice were randomized into control and experimental groups at the start of the experiment based on body weights, except for Fig. 2b, for which mice were matched for primary tumor size. Animal experiments were performed at least twice, with similar results observed, except for Figs. 1f, 2b,f,k, 3g–i, 4d,e,h, 6c–e, 7d and 8a–c and Extended Data Figs. 1f and 6b,d,f, which represent experiments performed once. No data were excluded from the analyses, except for Fig. 4h, in which one mouse (*Pdgfc* overexpression) did not develop a tumor due to a failed injection and therefore was excluded from metastasis analysis; Fig. 6c, in which one mouse (shNTC1) died under anesthesia during imaging before the experiment endpoint; Fig. 8b, in which a statistical outlier was identified (outlier analysis with GraphPad Prism (ROUT, *q* = 1%); outlier is highlighted, and *P* values with and without outlier are indicated); Fig. 8c, in which one mouse in the aged group treated with anti-PDGF-C antibody was excluded due to failed injection.

Statistics were performed using GraphPad Prism 8 apart from pathway analysis (IPA). All comparisons between two groups were made using a two-tailed, unpaired *t*-test unless the analysis did not pass a normality test (Shapiro–Wilk test), in which case, a two-tailed Mann–Whitney *U*-test was used. Equal variances were not formally tested. When more than two groups were compared, a one-way ANOVA test was performed with Holm–Sidak’s multiple-comparison test or a Kruskal–Wallis test with Dunn’s multiple-comparison test if the normality test was not passed (Shapiro–Wilk test). When there were two independent variables and multiple groups, a two-way ANOVA was performed with Sidak’s test for multiple comparisons or multiple *t*-tests for grouped analysis. Correlation analysis was performed using a two-tailed Pearson’s correlation test with *R*^2^ values shown. The box plots in Figs. 1d and 3e and Extended Data Figs. 1e and 2c show median (center line) and 25th–75th quartiles with the whiskers at minimum and maximum.

### Reporting summary

Further information on research design is available in the Nature Portfolio Reporting Summary linked to this article.

## Supplementary information

Supplementary Information Supplementary Tables 1–6 and Supplementary Figs. 1 and 2

Reporting Summary

## Data Availability

The mammary tumor cell line and young and aged lung RNA-seq datasets are deposited in the Sequence Read Archive under the accession number PRJNA822368. The dataset for RNA-seq of rPDGF-C treatment of MRC5 fibroblasts is deposited in the Sequence Read Archive under the accession number PRJNA895434. RNA-seq data from the METABRIC dataset were downloaded from cBioPortal^57,58^. RNA expression data for 57 human breast cancer cell lines were downloaded from the Broad Institute (CCLE). Non-cancerous lung gene expression data (GSE23546) and gene expression data for C57BL/6 mice treated with bleomycin (GSE40151) were downloaded from GEO. All other data supporting the findings of this study are available from the corresponding author on reasonable request. Source data are provided with this paper.

## Source data

Source Data Source data file with tabs for all figures and Extended Data figures and tabs for individual figure panels.
Source Data Fig. 5 Unprocessed western blots from Fig. 5.

## Code availability

Open-source software was used to analyze data in this study. Details of software versions and parameters are specified in relevant sections in the Methods.
