# Supplementary material for: Age-associated microenvironmental changes highlight the role of PDGF-C in ER+ breast cancer metastatic relapse
Source: Nat Cancer. 2023 Mar 13;4(4):468–84. doi: 10.1038/s43018-023-00525-y (PMC10132974; doi:10.1038/s43018-023-00525-y)
Supplement: Supplementary file 1 — Supplementary Tables 1–6 and Supplementary Figs. 1 and 2 [file 43018_2023_525_MOESM1_ESM.pdf]

nature cancer

Article https://doi.org/10.1038/s43018-023-00525-y

Age-associated microenvironmental changes highlight the role of PDGF-C in ER^+ breast cancer metastatic relapse

In the format provided by the authors and unedited

Supplementary Information

Supplementary Tables 1-6

Supplementary Table 1. | Antibodies and dilutions

Supplementary Table 2. | shRNA glycerol stocks

Supplementary Table 3. | RT-qPCR Taqman probes

Supplementary Table 4. | TSAE1 tumour signature

Supplementary Table 5. | Fibroblast activation signature

Supplementary Table 6. | Fibrosis Signature

Supplementary Figures

Supplementary Figure 1. | Example of FACS gating strategy to sort mCherry+ tumour cells from tissues.

Supplementary Figure 2. | Example of FACS gating strategy to sort cell populations from lungs.

Supplementary Table 1. | Antibodies and dilutions

Antibody	Product code	Company	Dilution	Application
Anti-mouse CD16/CD31 (Fc receptor block)	14-0161-82	eBioscience	1:100	FACS
CD140a APA5 (BV605 conjugated)	135916	BioLegend	1:200	FACS
CD31 390 (BV711 conjugated)	102449	BioLegend	1:400	FACS
CD326 G8.8 (APC/Cy7 conjugated)	118218	BioLegend	1:250	FACS
CD45 30-F11 (PE Cy7 conjugated)	103114	BioLegend	1:500	FACS
F4/80 Cl:A3-1 (AF647 conjugated)	MCA497A647	Bio-Rad	1:100	FACS
Actin (Smooth Muscle) (1A4)	A2547	Sigma	1:5,000	IF
Endomucin (V7C7)	sc-65495	Santa Cruz	1:1,000	IF
Endosialin (P13)	-	In-house	1:500	IF
HMGA2	PA521320	Thermo Fisher Scientific	1:300	IF
Lamin A + Lamin C [EPR4100]	ab108595	Abcam	1:1,000	IF
PDGF-C	TA351509	Origene	1:100	IF
Goat-anti-Mouse-IgG2a-488	A21131	Thermo Fisher Scientific	1:1,000	IF
Goat-anti-Rabbit-Ig-488	A11134	Thermo Fisher Scientific	1:1,000	IF
Goat-anti-Rabbit-Ig-555	A21429	Thermo Fisher Scientific	1:1,000	IF
Goat-anti-Rat-555	A21434	Thermo Fisher Scientific	1:1,000	IF
Actin (Smooth Muscle) (1A4)	M0851	Agilent DAKO	1:800	IHC
Actin (Smooth Muscle) (1A4)	M0851	Agilent DAKO	1:1,600	IHC + ISH
ERalpha 6F11	NCL-L-ER-6F11	Leica Biosystems	1:40	IHC
F4/80 Cl:A3-1	MCA497	Bio-Rad	1:100	IHC
Firefly luciferase	ab181640	Abcam	1:100	IHC
Lamin A + Lamin C (human) [EPR4100]	ab108595	Abcam	1:750	IHC
PDGF-C	AF1447	Bio-Techne	20 μg	*In vivo*
Goat IgG control	AB-108-C	Bio-Techne	20 μg	*In vivo*

Akt	9272	Cell Signaling	1:1,000	WB
p44/42 MAPK (Erk1/2) (137F5)	4695	Cell Signaling	1:1,000	WB
PDGF Receptor α (D1E1E) XP	3174	Cell Signaling	1:1,000	WB
Phospho-p44/42 MAPK (Erk1/2) (Thr202/Tyr204) (D13.14.4E) XP	4370	Cell Signaling	1:2,000	WB
Phospho-Akt (Ser473)	9271	Cell Signaling	1:1,000	WB
Phospho-PDGF Receptor α (Tyr 754)	441008G	Thermo Fisher Scientific	1:1,000	WB
Phospho-S6 Ribosomal Protein (Ser235/236) (D57.2.2E) XP	4858	Cell Signaling	1:2,000	WB
S6 Ribosomal Protein (5G10)	2217	Cell Signaling	1:1,000	WB
Vinculin [EPR20407]	ab219649	Abcam	1:1,000	WB
Goat anti-Rabbit IgG (H+L)-HRP	ab205718	Abcam	1:10,000	WB

FACS, Fluorescence activated cell sorting; IF, immunofluorescence; IHC, immunohistochemistry; ISH, *in situ* hybridisation; WB, Western blotting

Supplementary Table 2. | shRNA glycerol stocks (Sigma)

Product code	Target	Name
SHC002	Non-targeting control	shNTC1
SHC202	Non-targeting control	shNTC2
TRCN0000313940	*Pdgfc*	shPdgfc1
TRCN0000317595	*Pdgfc*	shPdgfc5
TRCN0000118191	*PDGFC*	shPDGFC2
TRCN0000331695	*PDGFC*	shPDGFC5

Supplementary Table 3. | RT-qPCR Taqman probes (Applied Biosystems)

Gene	Product code
B2m	Mm00437762_m1
Ipo8	Mm01255158_m1
Ubc	Mm01201237_m1
18s	Hs99999901_s1
B2M	Hs99999907_m1
IPO8	Hs00183533_m1
UBC	Hs00824723_m1
Esr1	Mm00433149_m1
Greb1	Mm00479269_m1
Pdgfa	Mm01205760_m1
Pdgfb	Mm00440677_m1
Pdgfc	Mm00480205_m1
Pdgfd	Mm00546829_m1
Pdgfra	Mm00440701_m1
PDGFA	Hs00234994_m1
PDGFB	Hs00966522_m1
PDGFC	Hs00211916_m1
PDGFD	Hs00228671_m1
PDGFRA	Hs00998018_m1

**Supplementary Table 4. | TSAE1 tumour signature**
B4galnt3
Bcat1
Cldn9
Clec2f
Csf3
Cwh43
Dlx2
Dmrta2
Dynap
Fosl1
Gjb4
Gm14137
Gpa33
Hmga2
Hoxa10
Il24
Klk6
Krt14
Krt16
Lypd3
Mettl7b
Mmp10
Nol4
Pax3
Pax6
Pkp1
Prl2c2
Ptpmt1
Rgs20
Rnf183
Serpinb5
Syt8
Tmprss11e
Wnt10a
Zic2

Supplementary Table 5. | Fibroblast activation signature

Acta2	*Egfr*	*Myc*	*Sgpl1*
Akap12	*Egr1*	*Ndufs4*	*Sirt6*
Akt1	*Emd*	*Nf1*	*Ski*
Anxa2	*Ereg*	*Ngfr*	*Slc8a1*
Appl1	*Esr1*	*Nlrc3*	*Sphk1*
Appl2	*Fap*	*Nov*	*Tgfb1*
Aqp1	*Fbxo4*	*Nupr1*	*Thbs1*
Arhgap4	*Fer*	*Pak1*	*Thy1*
Arhgef7	*Fgf10*	*Pak3*	*Tmem156*
Arid5b	*Fgf2*	*Parp1*	*Tmem201*
B4galt7	*Fgr*	*Parp10*	*Tnc*
Bag4	*Fn1*	*Pawr*	*Tns1*
Bax	*Fos*	*Pdcd4*	*Trim32*
Bmi1	*Fosl2*	*Pdgfa*	*Trp53*
Btc	*Fth1*	*Pdgfb*	*Trp53inp1*
Car12	*Fxyd3*	*Pdgfc*	*Ulk3*
Ccl2	*Gas6*	*Pdgfd*	*Wapl*
Ccna2	*Gclc*	*Pdgfra*	*Wdpcp*
Ccnb1	*Gclm*	*Pdgfrb*	*Wnt1*
Cd300a	*Gem*	*Pdlim1*	*Wnt2*
Cd74	*Gna12*	*Pex2*	*Wnt5a*
Cdc6	*Gstp1*	*Pip5k1a*	*Wnt7b*
Cdc73	*Has1*	*Pla2g1b*	*Zfand5*
Cdk4	*Hyal2*	*Plau*	*Zmiz1*
Cdk6	*Igf1*	*Pml*	*Zmpste24*
Cdkn1a	*Il17ra*	*Pparg*	
Cln3	*Ilk*	*Prkce*	
Col6a1	*Iqgap1*	*Prkdc*	
Col6a2	*Itgb1*	*Prr5l*	
Coro1c	*Itgb1bp1*	*Ptk2*	
Creb1	*Jun*	*Rab3b*	
Ctc1	*Krt17*	*Rb1*	
Cthrc1	*Lamtor2*	*Rcc2*	
Ctnnb1	*Lep*	*Rffl*	
Ctsk	*Lig4*	*Rgcc*	
Cygb	*Med25*	*Rnaseh2b*	
Dab2ip	*Med31*	*Rps6ka1*	
Dach1	*Mif*	*S100a4*	
Ddr2	*Mmp9*	*S100a6*	
Dhx9	*Morc3*	*Sdc4*	
Dmtn	*Mta2*	*Sesn2*	
E2f1	*Myb*	*Sfrp1*	

Supplementary Table 6. | Fibrosis Signature

Acvrl1	*Gusb*
Adam9	*Hpgd*
Arrb1	*Igf1*
Axl	*Ikbkb*
Bax	*Isyna1*
C3ar1	*Itgb3*
C6	*Itm2c*
Cav2	*Kcnn4*
Cavin2	*Lama3*
Ccr1	*Lgmn*
Cd68	*Lifr*
Cdc14b	*Loxl2*
Cdkn1a	*Lrrc32*
Cdon	*Masp1*
Col14a1	*Mme*
Col1a1	*Mmp12*
Col1a2	*Mmp16*
Col4a2	*Mmp8*
Col5a1	*Ms4a2*
Col5a3	*Myd88*
Cpxm2	*Ncam1*
Csf1r	*Ncf1*
Ctsb	*Osbpl5*
Ctsd	*Peli1*
Ctsk	*Pros1*
Cxcl12	*Ptpn1*
Dclk1	*Tgfbr2*
Dpysl3	*Thbs2*
Emp2	*Timp1*
Erp29	*Tmem100*
Evl	*Tpsb2*
Fabp4	*Trib3*
Fap	*Tspan12*
Fasn	*Vegfa*
Fgg	
Fhl2	
Flt1	
Fst	
Fzd5	
Gdf15	
Grem1	

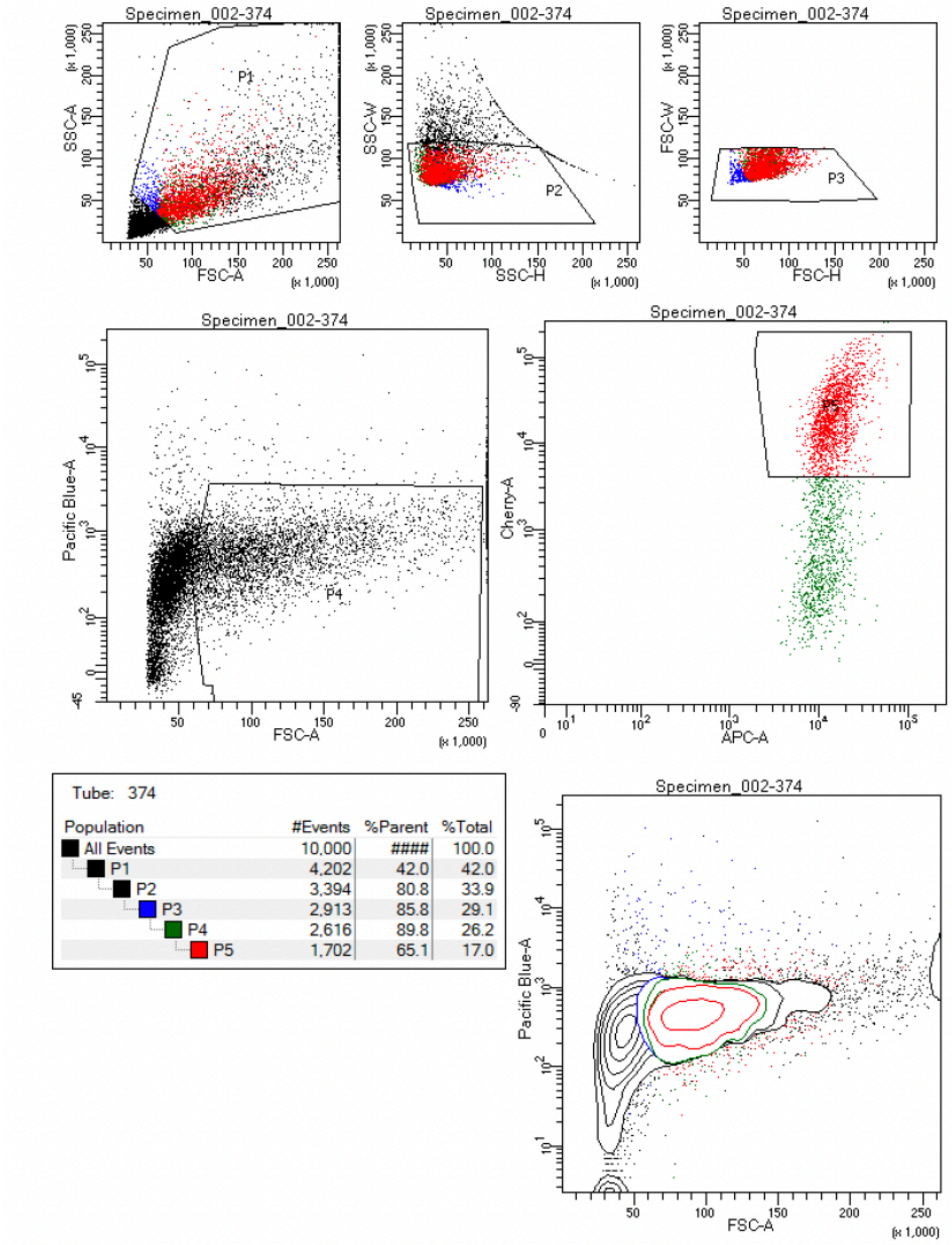

Population	#Events	%Parent	%Total
All Events	10,000	####	100.0
P1	4,202	42.0	42.0
P2	3,394	80.8	33.9
P3	2,913	85.8	29.1
P4	2,616	89.8	26.2
P5	1,702	65.1	17.0

Supplementary Figure 1. | Example of FACS gating strategy to sort mCherry+ tumour cells from tissues. Associated with Fig. 3i,j. Samples were gated to exclude cellular debris (FSC-A/SSC-A) and then SSC-H/SSC-W and FSC-H/FSC-W gates were used to discriminate doublets. DAPI was used to identify the live cell population. The tumour cell line (untagged and mChLuc2-tagged) cultured *in vitro* was used to set the mCherry gate.

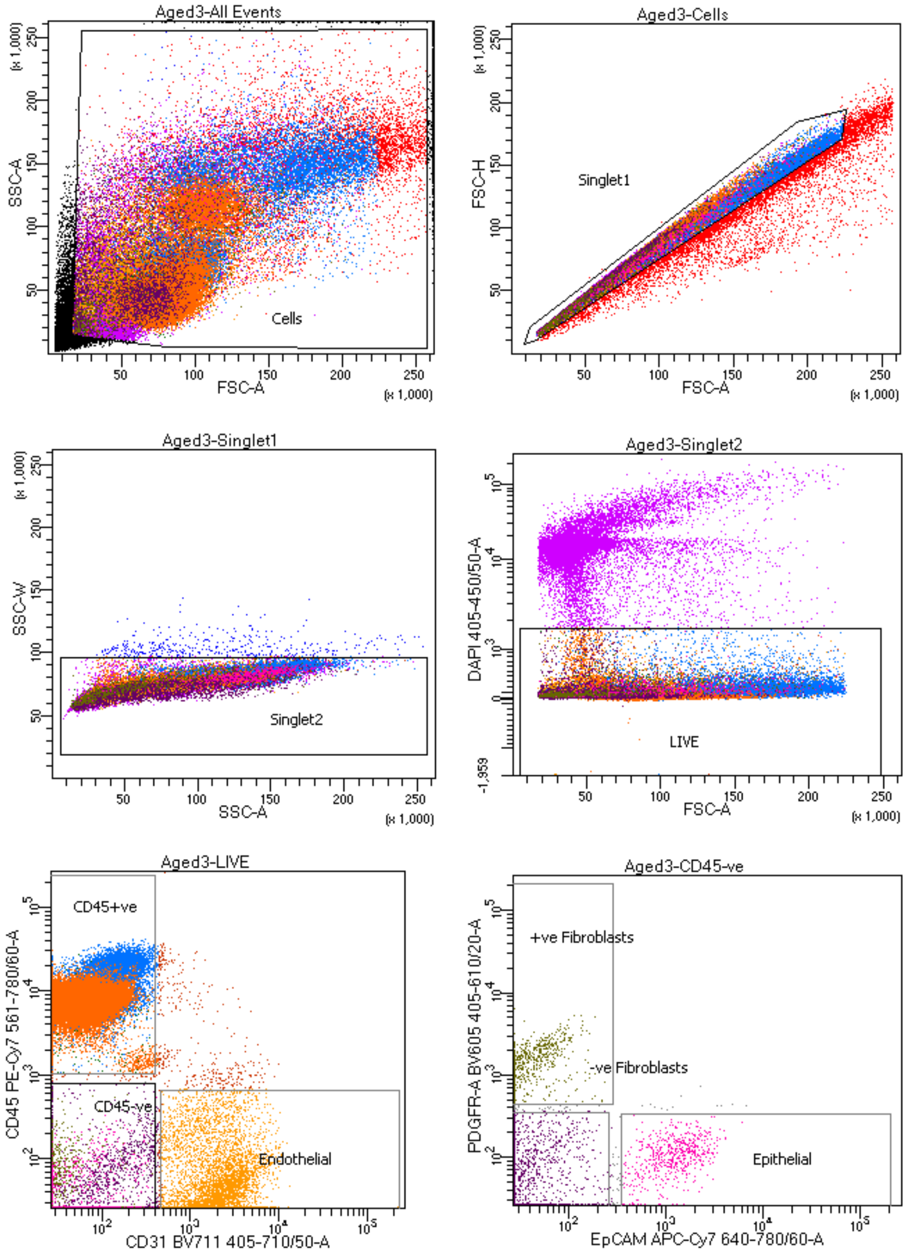

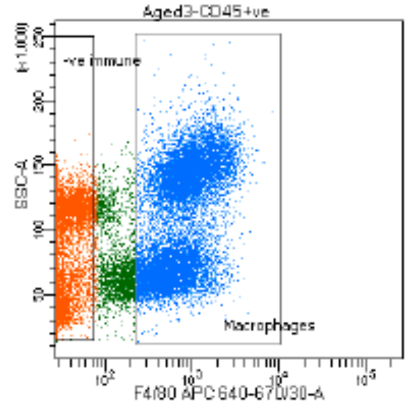

Tube: Aged3

Population	#Events	%Parent	%Total
All Events	109,196	####	100.0
Cells	100,000	91.6	91.6
Singlet1	93,876	93.9	86.0
Singlet2	93,503	99.6	85.6
LIVE	78,703	84.2	72.1
Endothelial	10,780	13.7	9.9
CD45-ve	5,340	6.8	4.9
-ve Fibroblasts	3,651	68.4	3.3
+ve Fibroblasts	1,009	18.9	0.9
Epithelial	561	10.5	0.5
CD45+ve	61,864	78.6	56.7
Macrophages	13,317	21.5	12.2
-ve immune	46,521	75.2	42.6

Supplementary Figure 2. | Example of FACS gating strategy to sort cell populations from aged lung. Associated with Extended Data Fig. 3c. Samples were gated to exclude cellular debris (FSC-A/SSC-A) and then SSC-A/SSC-W and FSC-A/FSC-W gates were used to discriminate doublets. DAPI was used to identify the live cell population. Cell populations were sorted as follows: CD45+ F4/80- (F4/80 negative immune cells); CD45+ F4/80+ (macrophages); CD45- CD31+ (endothelial cells); CD45- CD31- EpCAM+ (epithelial cells); CD45-, CD31-, EpCAM-, PDGFRα- (PDGFRα- fibroblasts); CD45-, CD31-, EpCAM-, PDGFRα+ (PDGFRα+ fibroblasts).
