# Supplementary material for: Age-associated microenvironmental changes highlight the role of PDGF-C in ER+ breast cancer metastatic relapse
Source: Nat Cancer. 2023 Mar 13;4(4):468–84. doi: 10.1038/s43018-023-00525-y (PMC10132974; doi:10.1038/s43018-023-00525-y)
Supplement: Supplementary file 2 — Reporting Summary [file 43018_2023_525_MOESM2_ESM.pdf]

nature portfolio

Corresponding author(s): Clare M. Isacke

Last updated by author(s): 15/1/23

Reporting Summary

Nature Portfolio wishes to improve the reproducibility of the work that we publish. This form provides structure for consistency and transparency in reporting. For further information on Nature Portfolio policies, see our Editorial Policies and the Editorial Policy Checklist.

Statistics

For all statistical analyses, confirm that the following items are present in the figure legend, table legend, main text, or Methods section.

n/a	Confirmed	
☐	☒	The exact sample size (*n*) for each experimental group/condition, given as a discrete number and unit of measurement
☒	☐	A statement on whether measurements were taken from distinct samples or whether the same sample was measured repeatedly
☐	☒	The statistical test(s) used AND whether they are one- or two-sided<br>*Only common tests should be described solely by name; describe more complex techniques in the Methods section.*
☐	☒	A description of all covariates tested
☐	☒	A description of any assumptions or corrections, such as tests of normality and adjustment for multiple comparisons
☐	☒	A full description of the statistical parameters including central tendency (e.g. means) or other basic estimates (e.g. regression coefficient) AND variation (e.g. standard deviation) or associated estimates of uncertainty (e.g. confidence intervals)
☐	☒	For null hypothesis testing, the test statistic (e.g. *F*, *t*, *r*) with confidence intervals, effect sizes, degrees of freedom and *P* value noted<br>*Give P values as exact values whenever suitable.*
☒	☐	For Bayesian analysis, information on the choice of priors and Markov chain Monte Carlo settings
☒	☐	For hierarchical and complex designs, identification of the appropriate level for tests and full reporting of outcomes
☐	☒	Estimates of effect sizes (e.g. Cohen's *d*, Pearson's *r*), indicating how they were calculated

Our web collection on statistics for biologists contains articles on many of the points above.

Software and code

Policy information about availability of computer code

Data collection	No software was used for data collection.
Data analysis	GraphPad Prism (8.4.3), Fiji (Image J; 2.0.0-rc-54/1.51h), Living image software (PerkinElmer,4.7.3), QuantStudio Real-Time PCR Software (1.7.1), NDP.view2 (2.7.52), Image Lab (Bio-rad, 6.1), SH800 and BD FACSDiva (8.0.1) software, Ventana Discovery Ultra (VSS v12.5.4). For analysis of RNA-seq data: FastQC (v0.11.4), and as previously described (Jungwirth et al. 2021 Nat. Commun.). For human fibroblast RNA-seq, trim-galore v0.6.6, GRCh38 using STAR aligner v2.7.6a, R package edgeR's (v3.28.1) TMM method in R statistical programming environment (v3.6.0). Gene Set Enrichment Analysis (GSEA 4.1.0), Ingenuity pathway analysis (version 01-20-04), heatmaps generated on MeV (4.8.1), R packages FactoMineR_(v2.4) and factoextra_(v1.0.7) for principal component analysis.

For manuscripts utilizing custom algorithms or software that are central to the research but not yet described in published literature, software must be made available to editors and reviewers. We strongly encourage code deposition in a community repository (e.g. GitHub). See the Nature Portfolio guidelines for submitting code & software for further information.

Data

Policy information about availability of data

All manuscripts must include a data availability statement. This statement should provide the following information, where applicable:

- Accession codes, unique identifiers, or web links for publicly available datasets
- A description of any restrictions on data availability
- For clinical datasets or third party data, please ensure that the statement adheres to our policy

The RNA-seq datasets (Fig1-3) are deposited in the Sequence Read Archive (SRA) under the accession number PRJNA822368. The data for the human fibroblast

RNA-seq (Fig 5) is deposited in SRA (PRJNA895434). METABRIC dataset were downloaded from cBioportal. Expression data for human breast cancer cell lines were downloaded from the Broad Institute (CCLE). Non-cancerous lung gene expression data (GSE23546) and gene expression data for C57BL/6 mice treated with bleomycin (GSE40151) were downloaded from GEO. Details of publicly available datasets analysed, and references to the original publications, are also included in the section 'Human data set analysis'.

Field-specific reporting

Please select the one below that is the best fit for your research. If you are not sure, read the appropriate sections before making your selection.

☒ Life sciences ☐ Behavioural & social sciences ☐ Ecological, evolutionary & environmental sciences

For a reference copy of the document with all sections, see nature.com/documents/nr-reporting-summary-flat.pdf

Life sciences study design

All studies must disclose on these points even when the disclosure is negative.

Sample size	Sample sizes for in vivo metastasis experiments were between 4 and 8 mice per group which was based on laboratory experience (Jungwirth et al. 2021 Nat. Commun; Jungwirth et al. 2018 DMM; Jenkins et al. 2022). One exception was N=3 D2A1 tumours for Pdgfc expression (Fig. 3h) due to mouse availability. For the experiment with non-tumour bearing mice used to assess expression (RT-qPCR) following vehicle/ bleomycin treatment (Fig. 2h) N=3 per group was considered sufficient for analysis of expression in the naive lung due to the absence of variability in tumour growth. For syngeneic spontaneous metastasis assays we used 6 mice per group (a minimum of 5 for xenograft experiments) due to the variability in primary tumour growth and thus increased variability/ higher standard deviations. Exceptions to this were Fig 2b (EdU analysis; N=4 young, N=3 aged) where groups where matched for primary tumour size.<br>For in vitro studies, a minimum of triplicates was used. In vitro sample sizes were chosen based on previous experience for such experiments (Jungwirth et al. 2021 Nat. Commu;Jungwirth et al. 2018 DMM).
Data exclusions	Fig. 4h, one mouse (TSAE1-Pdgfc o/e) did not develop a tumour due to a failed injection and therefore was excluded from the metastasis analysis. Fig. 6c, one mouse (shNTC1) died under anaesthesia during imaging before the experiment endpoint. Fig. 8c, one mouse in aged anti-PDGF-C group due to failed injection. A statistical outlier was identified in Fig 8b (outlier analysis on GraphPad Prism (ROUT, q = 1%)) but was highlighted on the graph and P-values with and without outlier are indicated.
Replication	Animal experiments were repeated on at least one additional occasion with equivalent results except experiments shown in Fig. 1f, Fig. 2b,f,k, Fig. 3g-i, Fig. 4d,e,h, Fig. 6c-e, Fig. 7d, Fig. 8a-c, Extended Data Fig. 1f, Extended Data Fig. 6b,d,f, which were performed once. Fig. 1f was repeated with a second model (Extended Data Fig. 1f); Fig. 2b, metastasis phenotype was seen in other experiments, the EdU was just performed once due to availability of aged mice; Fig. 2f, similar results were observed in experimental metastasis assays (Fig. 2c); Fig. 2k and was performed with two independent controls/shRNAs; Fig. 3g, similar results were observed in a second model (Extended Data Fig. 6b) and findings were validated by RNAScope analysis (Fig. 3f); Fig. 3h and Extended Data Fig. 6d,f were performed with two ER- lines; Fig. 3i, findings were validated by RNAScope analysis (Fig. 3f) and metastatic burden quantification of other experiments (Fig. 2a, Extended Data Fig 3a); Fig. 4d was validation in a second model; Fig. 4h and 6e was validation of a phenotype observed with knockdown; Fig. 6c, was validated in a second model and performed using two independent shRNAs; Fig 7d, Pdgfc knock-down and imatinib (in controls) findings had been seen in a independent experiment only the shPdgfc + imatinib arm was performed once; Fig. 8a,b, are two independent ways of inhibiting PDGFC pathway with similar results observed. Of the remaining in vivo experiments performed only once (Fig. 4e, 6d, Fig. 7b (shPdgfc + imatinib arm) and 8c), Fig. 8c was due to lack of availability of aged mice, Fig. 4e, Fig. 6d, Fig. 7b (shPdgfc + imatinib arm) provide additional data to help support main findings of the paper and had sufficient biological repeats (mice) in the experiment to perform statistical analysis . All in vitro experiments were repeated on at least one additional occasion with equivalent results and/or validated with different cell lines.
Randomization	In all animal experiments mice were randomized based on individual mouse body weights at the start of the experiment apart from Fig. 2b where mice were matched for primary tumour size. For in vitro studies, no randomization was performed as cell lines used in this study were from a single preparation with no reason to believe that the spacial location in the plates/wells affected results.
Blinding	The investigators were not blinded for the allocation of groups during experiments or during in vitro or in vivo experiments as the samples or animals were marked and the same investigator set-up and performed the experiment. Fully blinded animal experiments were not possible due to personnel availability to accommodate such situations. For imaging studies investigators were not blinded during data collection as the same investigator set up and imaged the experiment but all imaging analysis and quantification was performed blinded.

Reporting for specific materials, systems and methods

We require information from authors about some types of materials, experimental systems and methods used in many studies. Here, indicate whether each material, system or method listed is relevant to your study. If you are not sure if a list item applies to your research, read the appropriate section before selecting a response.

Materials & experimental systems

n/a	Involved in the study
[ ]	[x] Antibodies
[ ]	[x] Eukaryotic cell lines
[x]	[ ] Palaeontology and archaeology
[ ]	[x] Animals and other organisms
[x]	[ ] Human research participants
[x]	[ ] Clinical data
[x]	[ ] Dual use research of concern

Methods

n/a	Involved in the study
[x]	[ ] ChIP-seq
[ ]	[x] Flow cytometry
[x]	[ ] MRI-based neuroimaging

Antibodies

Antibodies used

Information on the antibodies used are provided in Supplementary Table 1 (copied below).

Anti-mouse CD16/CD31 (Fc receptor block) 14-0161-82 eBioscience 1:100 FACS
CD140a APA5 (BV605 conjugated) 135916 BioLegend 1:200 FACS
CD31 390 (BV711 conjugated) 102449 BioLegend 1:400 FACS
CD326 G8.8 (APC/Cy7 conjugated) 118218 BioLegend 1:250 FACS
CD45 30-F11 (PE Cy7 conjugated) 103114 BioLegend 1:500 FACS
F4/80 Cl:A3-1 (AF647 conjugated) MCA497A647 Bio-Rad 1:100 FACS

Actin (Smooth Muscle) (1A4) A2547 Sigma 1:5,000 IF
Endomucin (V7C7) sc-65495 Santa Cruz 1:1,000 IF
Endosialin (P13) - In-house 1:500 IF
HMGA2 PA521320 Thermo Fisher Scientific 1:300 IF
Lamin A + Lamin C [EPR4100] ab108595 Abcam 1:1,000 IF
PDGF-C TA351509 Origene 1:100 IF
Goat-anti-Mouse-IgG2a-488 A21131 Thermo Fisher Scientific 1:1,000 IF
Goat-anti-Rabbit-Ig-488 A11134 Thermo Fisher Scientific 1:1,000 IF
Goat-anti-Rabbit-Ig-555 A21429 Thermo Fisher Scientific 1:1,000 IF
Goat-anti-Rat-555 A21434 Thermo Fisher Scientific 1:1,000 IF

Actin (Smooth Muscle) (1A4) M0851 Agilent DAKO 1:800 IHC
Actin (Smooth Muscle) (1A4) M0851 Agilent DAKO 1:1,600 IHC + ISH
ERalpha 6F11 NCL-L-ER-6F11 Leica Biosystems 1:40 IHC
F4/80 Cl:A3-1 MCA497 Bio-Rad 1:100 IHC
Firefly luciferase ab181640 Abcam 1:100 IHC
Lamin A + Lamin C (human) [EPR4100] ab108595 Abcam 1:750 IHC

PDGF-C AF1447 Bio-Techne 20 ug In vivo
Goat IgG control AB-108-C Bio-Techne 20 ug In vivo

Akt 9272 Cell Signaling 1:1,000 WB
p44/42 MAPK (Erk1/2) (137F5) 4695 Cell Signaling 1:1,000 WB
PDGF Receptor α (D1E1E) XP 3174 Cell Signaling 1:1,000 WB
Phospho-p44/42 MAPK (Erk1/2) (Thr202/Tyr204) (D13.14.4E) XP 4370 Cell Signaling 1:2,000 WB
Phospho-Akt (Ser473) 9271 Cell Signaling 1:1,000 WB
Phospho-PDGF Receptor α (Tyr 754) 441008G Thermo Fisher Scientific 1:1,000 WB
Phospho-S6 Ribosomal Protein (Ser235/236) (D57.2.2E) XP 4858 Cell Signaling 1:2,000 WB
S6 Ribosomal Protein (5G10) 2217 Cell Signaling 1:1,000 WB
Vinculin [EPR20407] ab219649 Abcam 1:1,000 WB
Goat anti-Rabbit IgG (H+L)-HRP ab205718 Abcam 1:10,000 WB

FACS, Fluorescence activated cell sorting; IF, immunofluorescence; IHC, immunohistochemistry; ISH, in situ hybridisation; WB, Western blotting

Validation

Antibodies were obtained commercially and were validated by the manufacturer (see validation statements below) apart from P13 (Endosialin) that was made in-house and was validated by staining of MCF7 cells transfected with vector or endosialin (MacFadyen, 2007, GEP), with additionally no staining observed in FFPE tissue from Endosialin KO mice; antibodies for IHC were verified in-house by Breast Cancer Now Toby Robins Research Centre Nina Barough Pathology Core Facility using a panel of human and mouse tissues positive and negative for the target (for the luciferase antibody lungs 1.5h after iv injection of TSAE1-mChLuc2 or TSAE1-untagged cells were used as positive or negative controls, respectively).

Company validation statements:

Abcam: 'How we validate our antibodies: a closer look at the standards we use in the validation of our antibodies.

Here you will find more information on what is carried out during our application-specific validation processes' ... (see https://www.abcam.com/primary-antibodies/how-we-validate-our-antibodies).

Agilent DAKO: https://www.agilent.com/en/product/immunohistochemistry/antibodies-controls/primary-antibodies/actin-%28smooth-muscle%29-%28concentrate%29-76542

BioLegend: 'All of our products undergo industry-leading rigorous quality control (QC) testing to ensure the highest level of performance and reproducible results. Each lot is compared to an internally established "gold standard" to maintain lot-to-lot consistency. We also conduct wide-scale stability studies to guarantee an accurate shelf-life for our products. Additionally, we test the majority of our products on endogenous cells rather than transfected or immortal cells that may overexpress the analyte...' (see https://www.biolegend.com/en-us/quality/quality-control).

Bio-rad: 'Bio-Rad conducts rigorous in-house testing to guarantee that our antibodies meet our internal benchmarks and perform in their designated applications as expected. Our stringent quality control process is recognized by ISO9001:2015 certification at our manufacturing sites in Kidlington, Oxfordshire, UK, and Puchheim, Germany' (see https://www.bio-rad-antibodies.com/our-antibody-validation-principles.html?JSESSIONID_STERLING=F5A79FCF95CD46E21344A26EBA92A8BC.ecommerce1&evCntryLang=UK-en&EU_COOKIE_PREFS=000&cntry=UK&thirdPartyCookieEnabled=true).

Bio-techne: 'Our R&D Systems brand has been the leading antibody manufacturer for the past 30 years, using the best production standards and quality control specifications in the industry to develop antibodies scientists trust to exhibit high specificity and perform consistently every time. In addition to our established validation procedures, we also implement the 5 Pillars of Antibody Validation...' (see https://www.bio-techne.com/reagents/antibodies/antibody-validation).

Cell Signaling: 'To ensure product performance, we validate all of our antibodies, in-house, in multiple research applications' (see https://www.cellsignal.co.uk/about-us/our-approach-process/cst-antibody-performance-guarantee).

Invitrogen (ThermoScientific, eBioSciences): 'Invitrogen antibodies are currently undergoing a rigorous two-part testing approach Part 1—Target specificity verification - This helps ensure the antibody will bind to the correct target. Our antibodies are being tested using at least one of the following methods to ensure proper functionality in researcher's experiments'... (see https://www.thermofisher.com/uk/en/home/life-science/antibodies/invitrogen-antibody-validation.html)

Leica: https://shop.leicabiosystems.com/en-gb/pid-ER-6F11-L-CE_gl=1*7upcfo*_ga*MTU4OTE2NzQ2Mi4xNjczODUwMzEy*_ga_10W45KVS7Z*MTY3Mzg3NDE0OC4yLjEuMTY3Mzg3NDY5OS4wLjAuMA..

Origene: 'Specificity is one of the most important attributes of antibodies. At OriGene, we have been working hard to validate specificities of our antibodies. Validation Methods: Predicted band detected in Western blot analysis; Independent antibody strategies; 10k protein chip; Western blot using Knockout cell lysates...' (see https://www.origene.com/products/antibodies/quality).

Santa Cruz: ' Primary antibodies directed to mammalian target proteins have been characterized for reactivity against mouse, rat and human proteins' (see https://www.scbt.com/p/endomucin-antibody-v-7c7?gclid=Cj0KCQiAiJSeBhCCARIsAHnAzT8RJNnzQgHgQKPuiyzeqBbJPcvyGTMmxij1mVp7Qvic7Xu8VGRWt0waAnNXEALw_wcB)

Sigma: 'Delivering high-quality antibodies requires us to perform rigorous specificity and sensitivity testing in order to provide our customers with reliable tools that generate consistent results. We routinely perform standard validation processes across our antibody portfolio. Our standard antibody validation processes include verification for each recommended immunodetection application. Each of the thousands of antibodies in our portfolio are certified through our standard validation process to ensure quality and reproducibility' (see https://www.sigmaaldrich.com/GB/en/products/protein-biology/antibodies/enhanced-validation-ab)

Eukaryotic cell lines

Policy information about cell lines

Cell line source(s)	TSAE1 (TS/A-E1), HRM1, EMT6 and F311 cells were provided by Lalage Wakefield with permission from the scientists (Carla De Giovanni, Jean Zhao, Sara Rockwell and Daniel Alonso, respectively) that developed the cell lines. D2A1 and D2OR cells were from Ann Chambers laboratory stocks. D2A1-m1 and D2A1-m2 metastatic sublines were generated previously (Jungwirth et al. 2018 DDM). ZR-75-1, 10T1/2, 3T3, IMR90, 4T1 and HEK293T cells were from ATCC. AT-3 cells were provided by Christophe Paget and Daphnée Soulard (Institute Pasteur de Lille) and were originally from the lab that isolated them (Stewart et al, 2007 and 2009, J Immunol.). GFP-positive-CAFs were generated previously from 4T1-tumour-bearing BALB/c Ub-GFP mice (Jungwirth et al., 2021; Jungwirth et al. 2021 Nat. Commun). Young (BALB-5013 and C57-6013) and aged (A57-6013, two independent batches from 58-78 week-old mice) mouse primary lung fibroblasts were from Cell Biologics. GFP positive-CAFs were generated previously from 4T1-tumour-bearing BALB/c Ub-GFP mice.
Authentication	Human cell lines were tested using short tandem repeat.
Mycoplasma contamination	Cells were routinely tested for mycoplasma and tested negative.
Commonly misidentified lines (See ICLAC register)	No commonly misidentified cell lines were used in the study.

Animals and other organisms

Policy information about studies involving animals; ARRIVE guidelines recommended for reporting animal research

Laboratory animals	6-8 week-old female BALB/c, FVB, C57BL/6 and NSG mice were purchased from Charles River. The aged mice were aged in-house or purchased from Charles River.
Wild animals	No wild animals were used in this study.
Field-collected samples	No field-collected samples were used in this study.
Ethics oversight	All animal work was carried out under UK Home Office Project Licence P6AB1448A and PP4856884(Establishment Licence X702B0E74) granted under the Animals (Scientific Procedures) Act 1986 and approved by the 'Animal Welfare and Ethical Review Body' at the Institute of Cancer Research (ICR) and Imperial College. For primary tumour experiments the maximum tumour size permitted by the licence (mean diameter 18 mm) was not breached. Primary tumour growth data is included in the source data file.

Note that full information on the approval of the study protocol must also be provided in the manuscript.

Flow Cytometry

Plots

Confirm that:

☒ The axis labels state the marker and fluorochrome used (e.g. CD4-FITC).

☒ The axis scales are clearly visible. Include numbers along axes only for bottom left plot of group (a 'group' is an analysis of identical markers).

☒ All plots are contour plots with outliers or pseudocolor plots.

☒ A numerical value for number of cells or percentage (with statistics) is provided.

Methodology

Sample preparation	For the generation of mChLuc2 cell lines, mCherry+ tumour cells were sorted two-three passages after viral transduction on a FACS ARIA III cell sorter. For sorting of CAF-tumour cell co-cultures, cells were collected after 48h and sorted on a Sony SH800. For tissues: lungs and tumours were dissociated with the mouse tumour dissociation or lung dissociation kit (Miltenyi Biotec; 130-096-730 or 130-095-927), collected in Buffer S and dissociated into single cell suspensions using the gentleMACS Octo Dissociator (Miltenyi) using programme 37C_m_TDK_2 or 37C_m_LDK_1, respectively. Red blood cell lysis was performed (BD Biosciences, 555899) followed by an FC block (Thermofisher Scientific, 14-0161-85). Single cells were stained for CD45, CD31, EpCAM, PDGFRα and F4/80 and DAPI for sorting of the different populations (Extended Data Fig. 4c), or CD45, CD31 and DAPI for enable exclusion of immune cells, endothelial cells and dead cells (sorting mCherry+ tumour cells). respectively. Cells from tissues were sorted on a FACS ARIA III cell sorter or a Symphony S6 cell sorter.
Instrument	Sony SH800 cell sorter, FACS ARIA III or a Symphony S6 cell sorter.
Software	SH800 and BD FACSDiva (8.0.1) or (9.5) software.
Cell population abundance	A purity check was routinely performed with purity >80%.
Gating strategy	Samples were first gated to exclude cellular debris (FSC-A/SSC-A) and then SSC-H/SSC-W and FSC-H/FSC-W or SSC-A/SSC-W and FSC-A/FSC-H to discriminate doublets. DAPI was used to identify the live cell population for sorting of in vivo samples. The gate for mCherry was set based on positive and negative controls (tagged and untagged cells growing in vitro, respectively) and the gate for GFP set based on the monocultures (GFP+ CAFs, GFP- tumour cells). For sorting the cell populations from the lungs: CD45+ F4/80- (F4/80- immune cells), CD45+ F4/80+ (macrophages), CD45-CD31+ (endothelial), CD45-CD31-EpCAM+ (epithelial), CD45-CD31-EpCAM-PDGFRA- (PDGFRA- fibroblasts), CD45-CD31-EpCAM-PDGFRA+ (PDGFRA + fibroblasts) (Extended Data Fig. 4c) or CD45-CD31-mCherry positive for the sorting of tumour cells (Fig 3k). Examples of the gating strategies are shown in the Supplementary Information file.

☒ Tick this box to confirm that a figure exemplifying the gating strategy is provided in the Supplementary Information.
