# Supplementary figures and images for: Age-associated microenvironmental changes highlight the role of PDGF-C in ER+ breast cancer metastatic relapse
Source: Nat Cancer. 2023 Mar 13;4(4):468–84. doi: 10.1038/s43018-023-00525-y (PMC10132974; doi:10.1038/s43018-023-00525-y)

## Western blots from Fig. 5b

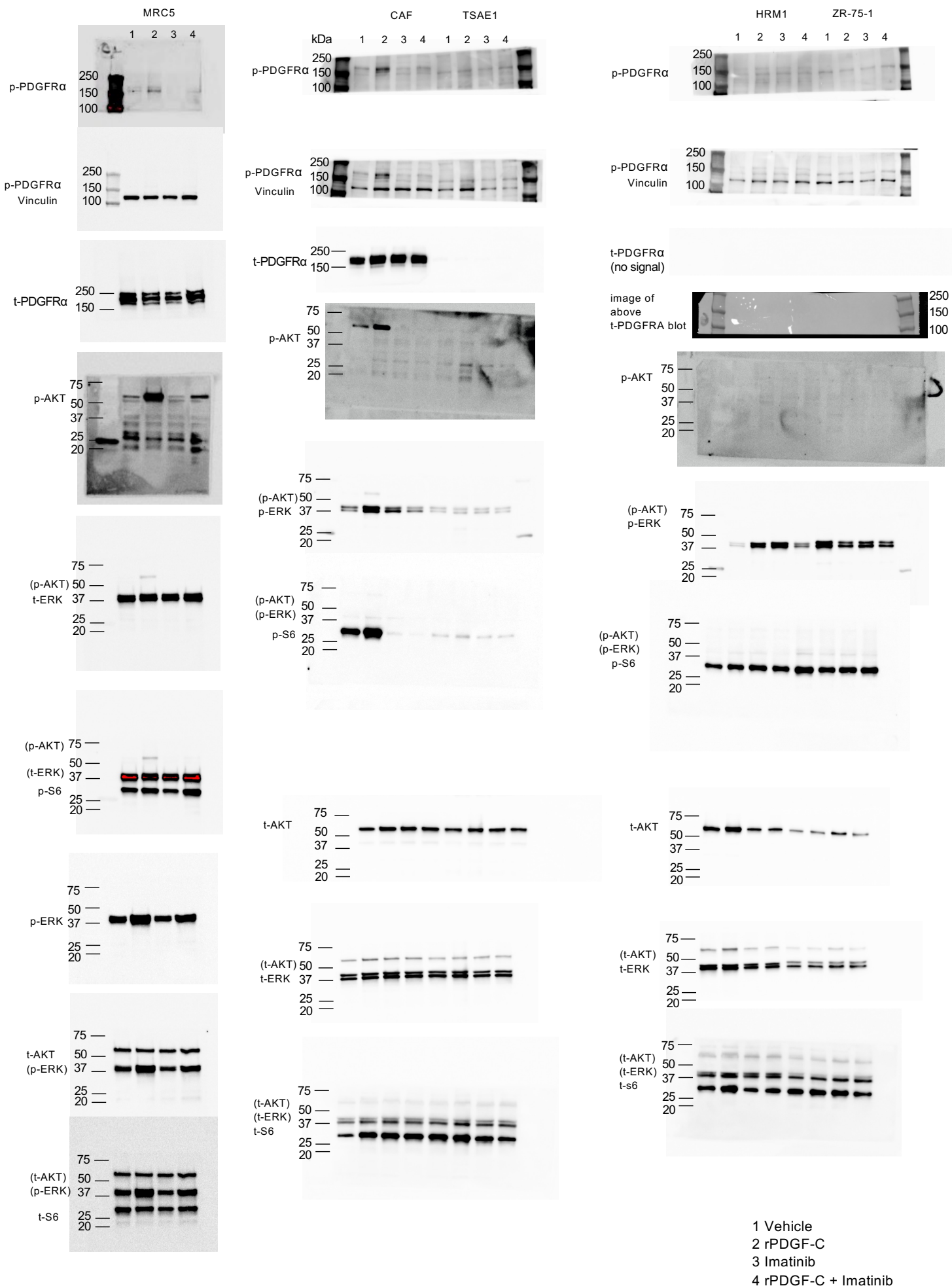

Supplement: Source Data Fig. 5 — Unprocessed western blots from Fig. 5. [file 43018_2023_525_MOESM4_ESM.pdf]
